# Combined strategy of dual-module cerium nanosystem composite extracellular vesicles regulate ROS in the tissue microenvironment to promote periodontitis recovery

**DOI:** 10.1016/j.mtbio.2025.102625

**Published:** 2025-12-04

**Authors:** Haozhe Ren, Peisheng Liu, Ziang Sun, Zhe Yu, Hao Guo, Xinyue Cai, Yihang Wei, Zihan Li, Meiling Wu, Xinyue Xu, Jing Wang, Kun Xuan

**Affiliations:** a School of Stomatology, Lanzhou University, Clinical Research Center for Oral Diseases of Gansu Province, Lanzhou, Gansu, 730000, China; b State Key Laboratory of Oral & Maxillofacial Reconstruction and Regeneration, National Clinical Research Center for Oral Diseases, Shaanxi Clinical Research Center for Oral Disease, Department of Preventive Dentistry, School of Stomatology, The Fourth Military Medical University, Xi’an, Shaanxi, 710032, China; c Department of Pharmaceutical Analysis, School of Pharmacy, The Fourth Military Medical University, Xi’an, Shaanxi, 710032, China

**Keywords:** Cerium-based nanoparticles, Single-atom cerium nanozymes, Extracellular vesicles (EVs), Antibacterial effect, Antioxidant stress, Periodontitis

## Abstract

Periodontal disease, a chronic inflammatory condition driven by dysbiotic biofilms and host immune dysregulation, lacks therapeutic strategies that simultaneously eliminate infection, resolve inflammation, and promote tissue regeneration. To bridge this gap, we designed a spatially compartmentalized dual-module nanosystem with ‘one cerium, dual functions’. Specifically, the antibacterial module utilizes hyaluronic acid (HA)-modified single-atom cerium nanozymes (Ce-N-C@HA) delivered to periodontal pockets. Upon HA degradation by bacterial hyaluronidase, the exposed catalytic sites generate hydroxyl radicals (·OH) via peroxidase like (POD-like) activity, enabling targeted pathogen eradication. Concurrently, the immunomodulatory module delivers *Lactobacillus reuteri*-derived extracellular vesicles (EVs) loaded with ultra-small CeO_2_ nanoparticles (CeO_2_-EV) into subgingival tissues. This module efficiently scavenges reactive oxygen species (ROS) and induces macrophage polarization to M2 anti-inflammatory phenotype, significantly alleviating the inflammatory microenvironment. By utilizing cerium’s dual functionalities which localized antibacterial action and reconstructs immune homeostasis, this system improves the periodontal microenvironment and promotes tissue regeneration, providing a novel strategy for nanotherapy in oral inflammatory diseases.

## Highlights

• Two different structures of cerium nanozymes (Ce-N-C and CeO_2_) constructed innovatively results in different activities.
• A novel Ce SAzyme (Ce-N-C) exhibits peroxidase-like activity, which is developed for antibacterial application.
• The CeO_2_-EV nanocomposite scavenges ROS and modulates immunity, promoting macrophage polarization to M2 phenotype.
• This “One cerium, dual functions” combined strategy provides a novel and efficient paradigm in periodontitis treatment.

## Graphical abstract

**Figure:**
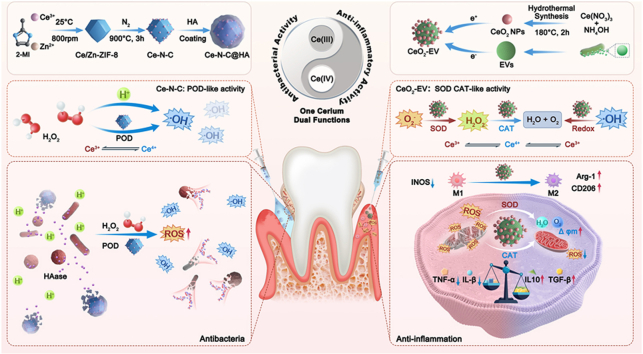

## 1 Introduction

Periodontitis is a plaque-induced, immune-mediated inflammatory disorder, with an estimated prevalence of 45–50 % worldwide [1]. Extensive evidence indicates that periodontitis could modulate the progression and clinical outcomes of multiple systemic diseases [[2], [3], [4]], including diabetes, cardiovascular disease, rheumatoid arthritis, and atherosclerosis. In terms of etiology, periodontitis is initiated by pathogenic oral bacteria [5], and pathological periodontal pockets serve as reservoirs for these microorganisms [6]. Further, multiple bacterial toxins, like the lipopolysaccharide (LPS) and gingipains, stimulate Toll-like receptors on immune cells, leading to the excessive production of reactive oxygen species (ROS) and activate the excessive inflammation. Different from common infectious diseases, the anatomical intervals of periodontal tissues form two relatively isolated unique microenvironments. The anaerobic biofilm microenvironment within the periodontal pocket and the immune and oxidative stress microenvironment around the subgingival tissues, which become the core reason for the difficulty in treating periodontitis [7,8]. Consequently, disrupting this pathogenic cycle necessitates a combined strategy of ROS regulation, targeted bacterial elimination and anti-inflammation, representing the optimal therapeutic pathway to restore periodontal homeostasis. However, current clinical treatments cannot simultaneously address all three aspects [[9], [10], [11]].

Nanozymes are a class of synthetic enzymes that combine the unique properties of nanomaterials with catalytic functions [[12], [13], [14], [15]]. Based on the different catalytic characteristics, redox-type nanozymes could be categorized into two types: one possesses catalase (CAT)-like activity or superoxide dismutase (SOD)-like activity, enabling ROS scavenging in antioxidant therapy [16]. The other exhibits peroxidase (POD)-like activity, catalyzing the conversion of hydrogen peroxide (H_2_O_2_) or oxygen (O_2_) into oxidizing hydroxyl radicals (·OH) via Fenton reaction [17]. This function demonstrates significant advantage for novel antimicrobial agents and tumor therapy [18]. By balancing ROS levels, both efficient antibacterial effects and reduced tissue oxidative stress could be achieved [[19], [20], [21], [22], [23]]. However, nanozymes normally could not fulfill simultaneously the antibacterial, antioxidant activity, and anti-inflammatory requirements inherent to periodontitis.

Cerium (Ce), a relatively stable lanthanide rare earth element, has provided an excellent opportunity to address the above issues. Cerium nanoparticles (Ce NPs) typically demonstrate superoxide dismutase and catalase activities, regulating oxidative stress in tissues, which has potential application in diseases [[24], [25], [26]]. The functional properties of Ce NPs are predominantly governed by their structural characteristics and surface morphology, such as size and the reversible Ce^3+^/Ce^4+^ [[27], [28], [29], [30]]. Traditional CeO_2_ adopts a face-centered cubic fluorite crystal structure, wherein Ce^4+^ ions reside in the octahedral interstitial sites, while O^2−^ ions occupy the tetrahedral interstitial sites. The proportion of Ce^3+^ is directly correlated with the oxygen vacancy concentration [31]. CeO_2_ exhibit potent antioxidant activity under physiological conditions, enabling reversible cycling between Ce^3+^/Ce^4+^ through oxygen vacancy-mediated binding. In addition, CeO_2_ has been also reported to have certain antibacterial properties due to POD-like activity [32]. Oxygen vacancy can reduce the rate-determining step energy barrier of its simulated POD catalysis and enhance the POD-like activity, which means containing an appreciable quantity of Ce^3+^ have higher POD-like activity [33]. These dual intrinsic constitute the most distinctive features of Ce NPs.

Single-atom Nanozymes (SAzymes) are a new type of nanozymes with single-atom dispersed active sites, and their activity originates from the dispersed single-atom structure and the optimized coordination environments. SAzymes exhibit remarkably enhanced enzyme-mimetic activities, which are attributed to the maximal utilization efficiency of metal atoms [[34], [35], [36]]. Generally, the M-N-C-type structure formed by nitrogen-coordinating atoms anchoring metal atoms is the main structural type of SAzymes [[37], [38], [39], [40]]. In this study, the Ce SAzymes(Ce-N-C) and ultrasmall CeO_2_ nanoparticles (<10 nm) were constructed. Strikingly, Ce-N-C developed herein did not exhibit enhanced CAT and SOD-like activities but demonstrated excellent POD-like activity, which can effectively promote ROS production, holding significant potential for antimicrobial applications. CeO_2_ can mimic antioxidant enzyme activity to scavenge ROS, exhibiting excellent antioxidant and free radical scavenging capabilities [41,42]. However, single-atom cerium nanozyme has rarely been reported, and the current research field is mainly in chemical and biological sensing [43].

Nanozymes are prone to rapid, uncontrolled release and are readily cleared from inflammatory sites, thereby restricting their anti-inflammatory efficacy. Based on these limitations of CeO_2_, extracellular vesicles (EVs) were also used to improve anti-inflammatory effects in this study. EVs with nanoscale (40–200 nm) lipid bilayer structures deriving from human commensal *Lactobacillus reuteri* (*L. reuteri*) can encapsulate bioactive proteins and nucleic acids, enabling them to dynamically mediate intercellular communication, modulate host immune responses, and promote tissue repair. Compared to the potential immunogenicity of live bacteria, EVs exhibit superior stability and enhanced targeting specificity to cells or tissues [[44], [45], [46], [47]]. However, whether CeO_2_ and EVs composite could better regulate the immune microenvironment remains unclear.

**Scheme 1:**
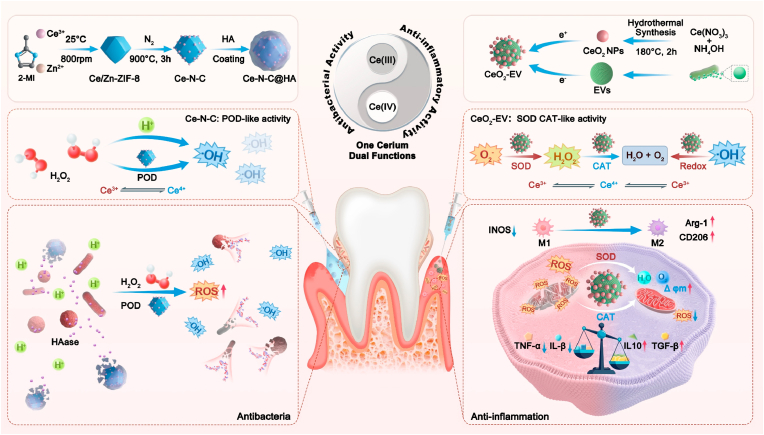
Systematic illustration of the construction of Ce-N-C@HA and CeO_2_-EV and the mechanisms underlying periodontal pathogen clearance by Ce-N-C@HA and periodontal inflammation inhibition by CeO_2_-EV.

To address these challenges of periodontitis, we integrated two structurally distinct cerium-based nanozymes with *L. reuteri-*derived EVs to engineer a dual-module cerium nanosystem for antibacterial and anti-inflammatory therapy against periodontitis. This platform achieves coordinated antibacterial action within the periodontal pocket and immunomodulation in the subgingival tissues immune microenvironment through regional regulation of ROS levels. In this system, hyaluronic acid (HA)-modified cerium single-atom nanozymes (Ce-N-C@HA) are injected into periodontal pockets. Subsequent degradation of the HA coating by bacteria-secreted hyaluronidase (HAase) exposes the Ce-N-C active core [48], triggering localized generation of ·OH and O_2_^−^ via POD-like activity and thereby effectively inhibiting bacterial proliferation. Complementarily, EVs were fused with CeO_2_ via electrostatic complexation to form CeO_2_-EV, which was subsequently injected into the subgingival tissues. This system effectively scavenges intracellular ROS levels and exerts anti-inflammatory functions, thereby promoting macrophage polarization toward the anti-inflammatory M2 phenotype. Collectively, this dual-functional cerium-based nanozyme strategy employs compartmentalized delivery, through distinct periodontal pocket and subgingival tissues injection, which achieves microenvironment-specific antibacterial effects and immunomodulation in periodontitis, thereby providing a novel approach for therapy of complex oral diseases (Scheme 1).

## 2 Materials and methods

### 2.1 Synthesis and characterization of Ce-ZIF-8 and Ce-N-C

Ce-ZIF-8 was prepared by dissolving 3.6945 g 2-methylimidazole (C_4_H_6_N_2_; M50850, Sigma-Aldrich, USA) in 80 mL anhydrous methanol (CH_3_OH; M116118, Aladdin, China) under sonication to obtain clear solution A. Separately, 1.66 g zinc nitrate hexahydrate (Zn(NO_3_)_2_·6H_2_O; Z111706, Aladdin, China) and 0.163 g cerium nitrate hexahydrate (Ce(NO_3_)_3_·6H_2_O; C431281, Aladdin, China) were dissolved in 40 mL methanol under sonication to yield solution B. Solutions A and B were combined and stirred at 25 °C for 24 h. The resulting product was collected by centrifugation at 8000 rpm, washed three times with methanol, and lyophilized for 24 h to afford Ce-ZIF-8. Subsequent pyrolysis at 900 °C for 3 h under N_2_ atmosphere produced single-atom cerium nanozyme Ce-N-C. Morphology and fine structure were examined by transmission electron microscopy (TEM, Hitachi HT-7700, Japan) and Scanning electron microscopy (SEM, Thermo Fisher, USA). Atomic-resolution imaging was performed with high-angle annular dark-field scanning transmission electron microscopy (HAADF-STEM, Thermo Scientific Themis Z, USA), and elemental mapping was acquired using high-resolution TEM (Thermo Scientific Talos F200X, USA). Metal loading was quantified by inductively coupled plasma optical emission spectroscopy (ICP-OES, Thermo Scientific iCAP 7600, USA). The elemental composition and chemical state of the surface using X-ray Photoelectron Spectroscopy (XPS; Thermo ESCALAB 250XI, USA). Crystalline structure was determined by X-ray diffraction (XRD, Bruker D8 Advance, Germany), and hydrodynamic size distribution was measured by dynamic light scattering (DLS, Malvern Zetasizer Nano ZS, UK). Peroxidase (POD) activity was assayed using a POD kit (BC0095, Solarbio, China).

### 2.2 Preparation of Ce-N-C@HA nanocomposite

The Ce-N-C@HA nanocomposite was prepared by adding 4 mL of Ce-N-C aqueous solution (0.2 mg/mL) into 4 mL of hyaluronic acid (HA; H131007, Aladdin, China) aqueous solution (8 mg/mL). After stirring at room temperature for 8 h, the mixture was centrifuged at 12,000 rpm, and the precipitate was washed three times with ultrapure water to obtain the final Ce-N-C@HA nanocomposites.

### 2.3 Bacterial strain culture and EVs extraction and identification

*L. reuteri* DSM 20016 used in the experiment was purchased from the Shanghai Bioresource Collection Center. The strain was inoculated on MRS agar medium and cultured in a 37 °C MGC anaerobic culture system (C-31, Mitsubishi Gas Chemical Company, Japan) under strictly anaerobic conditions (O_2_ < 0.1 %, CO_2_ 7–15 %).

The separation and extraction methods of EVs were performed as previously described [49]. Briefly, *L. reuteri* strains were cultured in MRS medium at 37 °C for 72 h, then harvested. Bacteria were removed by centrifugation at 5000 g for 20 min at 4 °C, and residual bacteria in the supernatant were filtered out using a 0.45 μm pore filter. The filtrate was then centrifuged at 150,000 g for 2.5 h at 4 °C using an ultracentrifuge (Beckman Coulter Optima L-80XP, USA) for separation. After discarding the supernatant, the solid precipitate was resuspended in PBS buffer and centrifuged again at high speed for 2.5 h. The EVs were collected and resuspended in PBS buffer. The protein concentration was determined using a BCA protein assay kit (P0012, Beyotime, China). The morphology of EVs was observed by TEM and SEM. The size and zeta potential of EVs were determined using DLS.

### 2.4 Preparation and Characterization of CeO_2_ nanoparticles

CeO_2_ nanoparticles were prepared via a hydrothermal method, yielding CeO_2_ precipitate which was subsequently vacuum-dried. Cerium nitrate hexahydrate and ammonia solution (NH_4_OH; A112080, Aladdin, China) were used as raw materials. First, Ce(NO_3_)_3_·6H_2_O was dissolved in deionized water to form a 0.1 M transparent solution, followed by the addition of ammonia water with stirring until a yellow dispersion was obtained. The pH of the reaction mixture was adjusted to 7.0 by alternating drops of nitric acid and ammonia water, and stirring was continued for 2 h. A 50 mL aliquot of the mixed solution was transferred into a stainless-steel autoclave lined with polytetrafluoroethylene, and the hydrothermal reaction was conducted at 180 °C for 12 h. After natural cooling to room temperature, the solid product was washed with deionized water and absolute ethanol, then freeze-dried for 12 h to obtain pale yellow CeO_2_ nanoparticles. The crystallinity, phase purity, crystal morphology, and particle size distribution of the product were systematically characterized using TEM. The elemental composition and chemical state of the surface using XPS. Functional groups were detected using Fourier Transform infrared spectroscopy (FTIR; Nicolet iS 50, USA). The zeta potential of CeO_2_ was determined using DLS.

### 2.5 Evaluation of In Vitro Antibacterial Activity of Ce-N-C@HA

*Staphylococcus aureus* (*S. aureus*, ATCC 25923), Methicillin-resistant *Staphylococcus aureus* (MRSA*,* ATCC 43300), and *Porphyromonas gingivalis* (*P. gingivalis*, ATCC 33277) were purchased from the Shanghai Bioresource Collection Center. *S. aureus* and MRSA were cultured on LB solid agar plates, while *P. gingivalis* was cultured on TSA solid agar plates in an MGC anaerobic culture system. Single colonies of the three bacterial strains were transferred from solid agar plates to corresponding liquid medium, cultured with shaking at 37 °C for 8 h, and the bacterial suspension concentration was adjusted to 1 × 10^7^ CFU/mL. Meanwhile, hyaluronidase (100U/ml; H4272, Sigma-Aldrich, USA) was added to the TSB liquid medium. Bacterial suspensions were treated with the following groups: (1) PBS control, (2) Ce-N-C, (3) 0.2 % H_2_O_2_, and (4) Ce-N-C@HA + H_2_O_2_. All treatment groups were cultured with shaking at 37 °C and 180 rpm, where the concentration of H_2_O_2_ was 0.2 %, and the concentrations of Ce-N-C and Ce-N-C@HA were 100 μg/mL. After 5 h of incubation, the bacterial suspension was diluted 10^5^-fold with liquid medium. Subsequently, 50 μL of the diluted bacterial suspension was spread on solid medium and cultured at 37 °C. The number and proportion of CFU were counted to evaluate the antibacterial effect of the cerium single-atom nanozyme.

### 2.6 Scanning electron microscopy characterization of bacterial samples

Bacterial suspensions from each group were collected by centrifugation, washed three times with sterile PBS, and then placed on 12 mm-diameter glass coverslips. They were subsequently fixed with 2.5 % glutaraldehyde at 4 °C for 2 h. The fixed samples were dehydrated through a gradient of ethanol solutions (30 %, 50 %, 70 %, 80 %, 90 %, 95 %, and 100 %) in sequence, immediately treated with 100 % hexamethyldisilazane for 40 min, and then dried overnight. Gold coating was applied using an ion sputter, and finally, morphological observation and analysis were performed using SEM.

### 2.7 Live/dead bacterial fluorescence staining analysis

Bacterial suspensions from each group were cultured at 37 °C for 5 h, then collected by centrifugation and washed with PBS. According to the manufacturer’s standard protocol, DMAO/PI dyes (C2030S, Beyotime, China) were added for staining at 37 °C in the dark for 15 min. After staining, the samples were washed three times with PBS to remove free dyes, and finally, fluorescence imaging analysis was performed using a confocal laser scanning microscope (CLSM; Olympus FV3000, Japan).

### 2.8 Cellular phagocytosis assay

EVs, CeO_2_ and CeO_2_-EV were incubated with Dil (468495, Sigma-Aldrich, USA) or FITC (F8071, Solarbio, China) in the dark for 2 h, and an equal volume of fetal bovine serum was added to terminate the reaction. The mixture was centrifuged at 150,000 g at 4 °C. After washing three times with PBS, the cells were fixed with 4 % paraformaldehyde at room temperature for 10 min and permeabilized with 0.1 % Triton X-100 at room temperature. Cells were incubated with Vari Fluor 594-Phalloidin or Vari Fluor 488-Phalloidin (MCE) in the dark for 20 min, followed by nuclear staining with DAPI (P0131, Beyotime Biotechnology, China) for 10 min. Observations were performed using a confocal laser scanning microscope (CLSM; Olympus FV3000, Japan).

### 2.9 Intracellular ROS level detection

The reactive oxygen species (ROS) detection kit (Beyotime Biotechnology, China) was used to evaluate the oxidative stress level in Raw264.7 macrophages. Specifically, Raw264.7 cells from different treatment groups were collected and incubated with 10 μM 2′,7′-dichlorodihydrofluorescein diacetate (DCFH-DA) probe at 37 °C in the dark for 20 min. Subsequently, the cells were washed twice with PBS to remove unbound probes, and counterstained with Hoechst 33342 (Sigma-Aldrich, 14533, China) at room temperature for 10 min for nuclear staining. ROS fluorescence intensity was quantitatively detected using a flow cytometer (Cytomics FC 500, Beckman Coulter, USA) and a confocal laser scanning microscope (CLSM; Olympus FV3000, Japan), with an excitation wavelength of 488 nm and an emission wavelength of 525 nm.

### 2.10 RNA isolation and qRT-PCR analysis

Total RNA was extracted using TRIzol LS reagent (Life Technologies, USA) and quantitatively detected with a Nanodrop 2000 UV–visible spectrophotometer to ensure the RNA concentration was within the range of 20–100 ng/μL. Subsequently, total RNA was reverse-transcribed into cDNA following the instructions of the High-Capacity cDNA Reverse Transcription Kit (Analytik Jena, USA). qRT-PCR analysis of cytokines were performed on a C1000 Touch PCR System (Bio-Rad, USA) using SYBR Green fluorescent dye.

### 2.11 ELISA analysis

Supernatants of Raw264.7 cells were collected and mixed with protease inhibitors in RIPA buffer, followed by incubation on ice. Subsequently, the mixture was centrifuged at 12,000 rpm for 10 min at 4 °C, and the liquid above the precipitate was collected. The protein concentrations of IL-10, TGF-1β, TNF-α, and IL-1β in macrophage culture supernatants were determined using commercially available ELISA kits (DuoSet, China) following the manufacturer’s instructions.

### 2.12 Flow cytometric analysis

Cells were seeded at a density of 0.5 × 10^6^ cells/mL in 10 cm uncoated culture dishes (10 mL medium per dish) and collected for detection after corresponding treatments. APC-fluorescently labeled antibodies were used to detect cell surface markers, specifically including APC-anti-iNOS antibody (696807, BioLegend, USA), APC-anti-CD206 antibody (141708, BioLegend, USA), and APC-anti-ARG1 antibody (165802, BioLegend, USA). Cells were incubated on ice in the dark for 30 min, washed twice with PBS, and subsequently analyzed using a flow cytometer (NovoCyte, ACEA Biosciences, USA).

### 2.13 Immunofluorescence analysis

Macrophages on glass coverslips were fixed with 4 % paraformaldehyde at room temperature for 15 min, permeabilized with 0.1 % Triton X-100 (Sigma-Aldrich, 93443) for 15 min, and then blocked with 5 % BSA at 37 °C for 1 h. Primary antibodies, including Anti-F4/80 antibody [CI: A3-1] - Macrophage Marker (ab6640, Abcam, USA), CD206/MRC1 (E6T5J) XP® Rabbit mAb (24595, Cell Signaling Technology, USA), Arginase-1 (D4E3M™) XP® Rabbit mAb (93668, Cell Signaling Technology, USA), and iNOS (D6B6S) Rabbit mAb (13120, Cell Signaling Technology, USA), were applied and incubated overnight at 4 °C. After washing three times with PBS, the cells were incubated with secondary antibodies Alexa Fluor™ 594 Donkey anti-Rat IgG (H + L) (1:200; Invitrogen, USA) and Alexa Fluor™ 488 Donkey anti-Rabbit IgG (H + L) (1:200; Invitrogen, USA) at 37 °C for 30 min. Nuclear staining was performed with DAPI (P0131, Beyotime Biotechnology, China) for 10 min, followed by mounting with coverslips. Images were captured using a confocal laser scanning microscope (CLSM; Olympus FV3000, Japan).

### 2.14 Western blot analysis

Cells were seeded in 6-well plates at a density of 5 × 10^5^ cells per well. When cell confluency reached approximately 70 %–75 %, they were stimulated with 1 μg/mL LPS (DY40109, Deeyee, China) for 12 h. The original medium was removed, and cells were washed twice with PBS. Subsequently, complete medium containing different intervention materials was added to each group of cells, and total proteins were collected after 24 h. Cells were lysed on ice using RIPA lysis buffer (Beyotime, China) supplemented with protease inhibitors (Roche, Cat. No. 04693132001), and protein concentration was determined using a BCA protein assay kit (Beyotime, China). A total of 20 μg of protein per sample was separated by SDS-polyacrylamide gel electrophoresis and transferred to polyvinylidene difluoride (PVDF) membranes (Millipore, Germany). The membranes were blocked with 5 % bovine serum albumin (BSA, MP Biomedicals, USA) at room temperature for 2 h, then incubated overnight at 4 °C with primary antibodies against iNOS (13120S, Cell Signaling Technology, USA), Arginase-1 (93668S, Cell Signaling Technology, USA), and β-actin (4967S, Cell Signaling Technology, USA). After washing with TBST buffer, the membranes were incubated with corresponding secondary antibodies at room temperature for 1 h. Finally, protein bands were visualized using a ChemiDoc chemiluminescence detection system (Bio-Rad, China).

### 2.15 Analysis of mitochondrial membrane potential

Mitochondrial membrane potential was determined using the JC-1 Mitochondrial Membrane Potential Detection Kit (C2006, Beyotime, China) following the standard protocol provided by the manufacturer. Specifically, cells were seeded at a density of 1 × 10^5^ cells per 20 mm glass-bottom confocal dish, stimulated with 1 μg/mL LPS (DY40109, Deeyee, China) for 12 h, then the original medium was removed and cells were washed once with PBS. Subsequently, complete medium containing different intervention materials was added to each group of cells. After washing once with PBS, 1 mL of 1 × JC-1 staining buffer was added for staining at 37 °C for 20 min. Cell images were acquired using a confocal laser scanning microscope (CLSM; Olympus FV3000, Japan).

### 2.16 Mitochondrial superoxide detection

Mitochondrial superoxide was determined using the Mitochondrial Superoxide Assay Kit (S0061S, Beyotime, China) following the standard protocol provided by the manufacturer. Specifically, cells were seeded at a density of 1 × 10^5^ cells per 20 mm glass-bottom confocal dish, stimulated with 1 μg/mL LPS (DY40109, Deeyee, China) for 12 h, then the original medium was removed and cells were washed once with PBS. Subsequently, complete medium containing different intervention materials was added to each group of cells. After washing once with PBS, 1 mL of MitoSO™ Red staining working solution was added for incubation at 37 °C for 20 min. Cell images were acquired using a confocal laser scanning microscope (CLSM; Olympus FV3000, Japan).

### 2.17 RNA sequencing

The total RNA from Raw264.7 cells treated with CeO_2_-EV or LPS (n = 3 per group) was extracted by the TRIzol Reagent. After determining the RNA concentration and purity, 1 μg of high-quality RNA per sample was used as input material for RNA sample preparations. Sequencing libraries were generated using the Hieff NGS Ultima Dual-mode mRNA Library Prep Kit for Illumina in accordance with the manufacturer’s recommendations, and unique index codes were added to attribute sequences to each sample. Libraries were sequenced on an Illumina NovaSeq platform to generate 150 bp paired-end reads, in accordance with the manufacturer’s instructions. Differentially expressed genes (DEGs) were identified by comparing the CeO_2_-EV group with the LPS group using the DESeq2 package in RStudio software (version 4.3.1). These DEGs were defined as those with a fold change ≥1.5 and a false discovery rate (FDR) < 0.05. The identified DEGs were subjected to further functional enrichment analysis based on the Gene Ontology (GO) database and Kyoto Encyclopedia of Genes and Genomes (KEGG) database.

### 2.18 Animals

Eight-week-old male C57BL/6 mice (purchased from the Experimental Animal Center of the Fourth Military Medical University, Xi’an) were used in this study. The mice were housed in a specific pathogen-free (SPF) environment with a 12 h/12 h light-dark cycle and provided with free access to food and water. All animal experimental procedures were strictly conducted in accordance with the *Guide for the Care and Use of Laboratory Animals* and approved by the Institutional Animal Care and Use Committee of Air Force Medical University.

### 2.19 Establishment of ligature-induced periodontitis model

An experimental periodontitis model was established in 8-week-old C57BL/6 mice. Specifically, 5-0 silk threads were pre-soaked in an anaerobic culture solution (1 × 10^9^ CFU/mL) of *Porphyromonas gingivalis*(ATCC 33277, SHMCC, China) for 24 h to enhance plaque colonization and inflammation induction. Subsequently, the threads were circumferentially ligated around the buccal gingival sulcus of the maxillary second molar (M2) under sterile conditions. After ligation, 10 μL of Ce-N-C@HA solution (100 μg/mL) containing 0.2 % H_2_O_2_ was injected into the deep periodontal pockets on 1 d, 4 d and 7 d. Besides, a 10 μL suspension of CeO_2_-EV complex (mass ratio 5:1, total concentration 50 μg/mL) was subcutaneously injected at the midpoint of the buccal gingival papilla of the maxillary second molar (injection depth 0.5 mm). All mice were sacrificed uniformly on 14 d for sample collection.

### 2.20 CBCT analysis

Samples were fixed in 4 % paraformaldehyde (PFA, Sigma-Aldrich, USA) at 4 °C for 24 h to ensure sufficient fixation. Subsequently, samples were scanned using CBCT (Siemens Inveon, Germany and PerkinElmer, USA) with the following parameters: spatial resolution of 18 μm, tube voltage of 50 kV, and tube current of 80 μA. CBCT data were imported into CTan for three-dimensional reconstruction and quantitative analysis. The measured indicators included: the distance from the buccal cementoenamel junction (CEJ) of the second molar to the alveolar bone crest (ABC); bone volume fraction (BV/TV), bone mineral density (BMD), trabecular number (Tb.N), trabecular thickness (Tb.Th), and trabecular separation (Tb.Sp) in the region between the first and second molars.

### 2.21 Histological and immunofluorescence analysis

Maxillary bone samples from mice were collected and fixed in 4 % paraformaldehyde (PFA, Servicebio, China) at 4 °C for 24 h, then transferred to 17 % ethylenediaminetetraacetic acid (EDTA, Servicebio, China) decalcifying solution for decalcification at 4 °C for 21 d, with daily replacement of the decalcifying solution during this period. After decalcification, samples were thoroughly rinsed with running water, dehydrated through a gradient of ethanol solutions (50 %, 70 %, 85 %, 95 %, and 100 %), and embedded in paraffin for serial sectioning (5 μm thickness). Sections were baked in a 65 °C oven for 3 h, dewaxed twice with xylene (30 min each time), and hydrated by immersion in 100 %, 90 %, 80 %, and 75 % ethanol sequentially (5 min each). Hematoxylin-eosin (H&E, Leica, Germany) staining, Masson staining (Masson, Baso, China), and tartrate-resistant acid phosphatase (TRAP, Solarbio, China) staining were performed following the manufacturers’ standard protocols. Microscopic images were captured using a research-grade whole-slide scanning system (Olympus VS200, Japan), and image analysis was conducted using Image-Pro Plus 6.0 software.

For immunofluorescence staining, sections were baked in a 65 °C oven for 3 h, dewaxed with xylene, hydrated through gradient ethanol, and gently rinsed with distilled water. Antigen retrieval was performed using Tris-EDTA Antigen Retrieval Solution (10 × , Solarbio) under heat for 2 min, followed by three washes in PBS buffer. Primary antibodies, including Anti-IL-1 beta antibody (ab319085, Abcam, USA), TGF Beta 1 Polyclonal antibody (21898-1-AP, Proteintech, China), CD206/MRC1 (E6T5J) XP® Rabbit mAb (24595, Cell Signaling Technology, USA), Arginase-1 (D4E3M™) XP® Rabbit mAb (93668, Cell Signaling Technology, USA), and iNOS (D6B6S) Rabbit mAb (13120, Cell Signaling Technology, USA), were applied for overnight. Secondary antibodies, Alexa Fluor™ 594 Donkey anti-Rat IgG (H + L) (1:200; Invitrogen, USA) and Alexa Fluor™ 488 Donkey anti-Rabbit IgG (H + L) (1:200; Invitrogen, USA), were added to the sections and incubated at 37 °C for 30 min. Nuclei were stained with DAPI (P0131, Beyotime Biotechnology). Stained sections were observed using a laser scanning confocal microscope (Nikon, Japan).

### 2.22 Animal *in vivo* imaging

For biodistribution analysis, CeO_2_-EV were labeled with the fluorescent lipophilic tracer DiR (Yeasen, China) and Ce-N-C@HA were labeled with the Cy7 (Yeasen, China). Both of them were detected for 72 h *in vivo.* The biodistribution imaging *in vivo* was obtained by IVIS Spectrum (PerkinElmer, USA).

### 2.23 Statistical analysis

All the experimental data were presented as the Mean ± Standard Deviation (SD) from at least three independent experiments. All statistical analyses were performed using GraphPad Prism 8.0 and Origin 2021 software. Significance was assessed by Student’s *t*-test for two group comparisons, and one-way ANOVA followed by the Tukey’s post-hoc test for multiple group comparisons. A *P*-value <0.05 was considered statistically significant.

## 3 Results and discussion

### 3.1 Synthesis and characterization of Ce-N-C

**Fig. 1:**
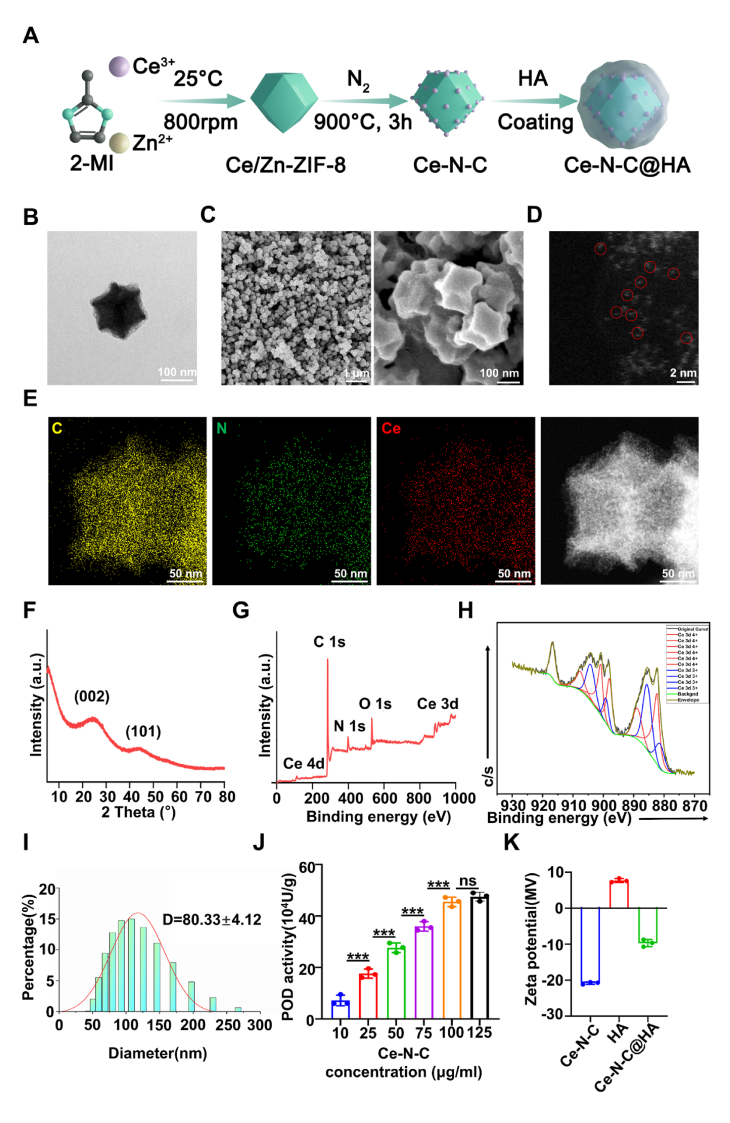
Synthesis and Characterization of Ce-N-C. (A) Schematic diagram of the Ce-N-C synthesis process. (B) TEM image of Ce-N-C. (C) SEM images of Ce-N-C. (D) HAADF-STEM image of Ce-N-C exhibiting dispersive single Ce atoms as bright dots (indicated by red circles). Bar: 2 nm. (E) EDS mapping images of Ce-N-C. (F) XRD pattern of Ce-N-C. (G) XPS images of Ce-N-C and (H) peak-differentiating of Ce 3d. (I) Hydrodynamic size of Ce-N-C. (J) POD activity of Ce-N-C. n = 3 per group. (K) Zeta potential of Ce-N-C, HA, and Ce-N-C@HA. n = 3 per group. Data are presented as Mean ± SD. *∗∗∗P* < 0.001; ns, no significance. (For interpretation of the references to colour in this figure legend, the reader is referred to the Web version of this article.)

Initially, Ce SAzyme was synthesized by a metal-organic framework (MOF) as the template (Fig. 1A). Specifically, Ce(NO_3_)_3_ molecules were encapsulated within a zeolitic imidazolate framework-8 (ZIF-8), yielding the cerium-doped framework Ce-ZIF-8. The successful *in situ* encapsulation within ZIF-8 was enabled by the average molecular diameter of Ce(NO_3_)_3_ (9.8 Å), which lies between the pore size (3.4 Å) and cavity size (11.6 Å) of the host ZIF-8 structure. Scanning electron microscopy (SEM) and transmission electron microscopy (TEM) images revealed a uniform rhombic dodecahedron morphology for the resulting Ce-ZIF-8 nanoparticles, with diameters ranging from 80 to 120 nm (Fig. S1A and B), consistent with previously reported ZIF-8 crystal structure [50]. Furthermore, X-ray diffraction (XRD) spectra of Ce-ZIF-8 and pristine ZIF-8 were found to be nearly identical (Fig. S1C), indicating that the incorporation of Ce(NO_3_)_3_ molecular guests did not alter the main crystal structure of the ZIF-8 framework. Subsequently, the Ce-ZIF-8 precursor underwent pyrolysis at 900 °C. During this thermal treatment, zinc atoms progressively evaporated, inducing skeletal decomposition of the framework. This facilitated the anchoring of Ce atoms onto the forming nitrogen-doped porous carbon (N-C) matrix, ultimately yielding single-atom Ce catalysts (Ce-N-C) with atomic dispersion. SEM and TEM analyses confirmed retention of a rhombic dodecahedral morphology with approximately 100 nm diameters and uniform surface structure (Fig. 1B and C). High-angle annular dark-field scanning transmission electron microscopy (HAADF-STEM) images revealed distinct isolated bright dots (Fig. 1D), attributable to the pronounced Z-contrast of atomic Ce against the C/N matrix, directly evidencing the atomic dispersion of Ce within the nitrogen-doped carbon scaffold. Energy-dispersive spectroscopy (EDS) further demonstrated the homogeneous spatial distribution of Ce, N, and C throughout the material (Fig. 1E). Quantitative analysis via inductively coupled plasma optical emission spectrometry (ICP-OES) determined a cerium content of 0.896 wt%. XRD patterns exhibited only the characteristic (002) and (101) diffraction peaks of graphitic carbon at 24° and 43°, respectively, with no detectable crystalline cerium phases, confirming the absence of metallic Ce or cerium-containing nanoparticles (Fig. 1F). X-ray photoelectron spectroscopy (XPS) analysis indicated a Ce^4+^/Ce^3+^ ratio of 38.53 % and 61.47 %. (Fig. 1G and H). Dynamic light scattering (DLS) showed a hydrated particle size of 80.33 ± 4.12 nm (Fig. 1I).

Compared to cerium oxide nanozymes, we discovered that Ce-N-C configuration exhibits distinct catalytic properties, primarily displaying POD-like activity, which is possibly related to the high Ce^3+^ ratio. To evaluate potential Fenton reaction activity in the synthesized Ce-N-C, its POD-like activity was quantified across concentrations ranging from 10 to 125 μg/mL. Enzyme activity demonstrated a concentration-dependent increase, plateauing between 100 and 125 μg/mL without statistical significance (Fig. 1J), confirming intrinsic POD-mimetic properties. This catalytic capability can effectively generate ROS, indicating potential antibacterial activity.

Zeta potential measurements showed values of −20.56 mV for Ce-N-C, +7.63 mV for HA, and −9.64 mV for Ce-N-C@HA (Fig. 1K). This significant shift in surface charge confirms the feasibility of electrostatic adsorption-driven assembly between Ce-N-C and HA. Given HA established role in active bacterial targeting, cellular proliferation, and adhesion enhancement, Ce-N-C was encapsulated within HA (Ce-N-C@HA) to augment biocompatibility. Cytotoxicity assessment against RAW264.7 cells via trypan blue exclusion revealed sustained cell viability exceeding 85 % at 12 h, 24 h, and 48 h, with cells maintaining viable morphology (Fig. S2A and B). Live/Dead staining showed that PDLCs and HOK cells viability remained above 90 % after 72 h exposure to Ce-N-C@HA, confirming favorable cytocompatibility (Fig. S2C-E). These results substantiate HA’s effectiveness in improving the biocompatibility profile of Ce-N-C.

### 3.2 Preparation and Characterization of CeO_2_-EV

**Fig. 2:**
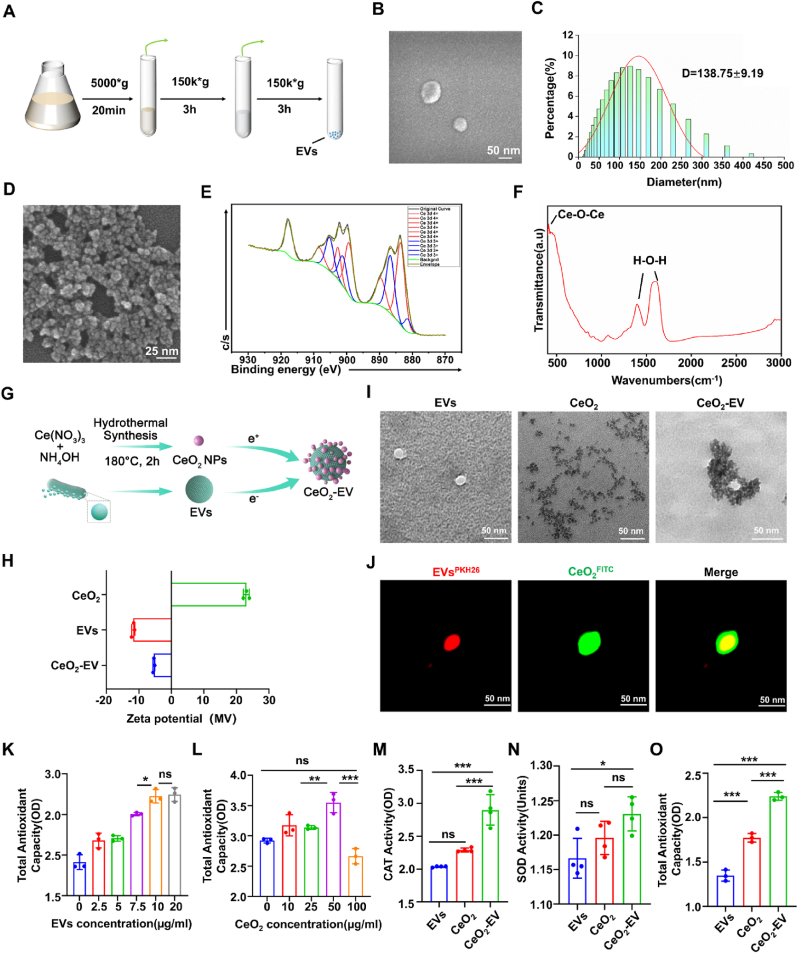
Preparation and Characterization of CeO_2_-EV. (A) Schematic diagram for the extraction process of EVs. (B) Representative TEM image showing the morphology of EVs. Bar: 50 nm. (C) DLS showing the distribution of EVs particle size. (D) Representative SEM image showing the morphology of CeO_2_. Bar: 25 nm. (E) XPS peak-differentiating image of Ce 3d in CeO_2_. (F) FTIR spectra of CeO_2_. (G) Schematic diagram of electrostatic adsorption between CeO_2_ and EVs. (H) Zeta potential and (I) representative TEM images of EVs, CeO_2_ and CeO_2_-EV. Bar: 50 nm. (J) Fluorescence schematic diagram: CeO_2_-EV. PKH26 and FITC label EVs and CeO_2_ respectively. Bar: 50 nm. (K, L) The quantification for the total antioxidant capacity of EVs and CeO_2_ at different concentrations. (M − O) The quantification for the CAT activity, SOD activity and total antioxidant capacity of EVs, CeO_2_ and CeO_2_-EV. Data are presented as Mean ± SD. *∗P* < 0.05; *∗∗P* < 0.01; *∗∗∗P* < 0.001; ns, no significance.

*L. reuteri* EVs were purified via ultracentrifugation (Fig. 2A). Bacterial growth reached the plateau phase at 24 h (Fig. S3A), while the BCA protein assay showed the highest level of EVs protein occurred after 72 h (Fig. S3B). SEM revealed spherical surface structures of EVs (Fig. 2B), with TEM further confirming their double-layered spherical membrane morphology (Fig. 2I). DLS analysis indicated an average hydrated particle size of 138.75 ± 9.19 nm (Fig. 2C), consistent with reported dimensions of bacterial membrane vesicles [51].

Ultra-small CeO_2_ was synthesized via a hydrothermal reaction using Ce(NO_3_)_3_·6H_2_O and ammonium hydroxide as precursors. SEM and TEM analyses revealed that the resulting CeO_2_ nanocrystals possessed a uniform size of 5–10 nm, with smaller particles exhibiting a tendency to assemble into nanoclusters (Fig. 2D–I). XPS analysis showed Ce^4+^ and Ce^3+^ contents of 65.01 % and 34.99 %, respectively, corresponding to a Ce^4+^/Ce^3+^ ratio of 1.82 (Fig. 2E). Fourier transform infrared spectroscopy (FTIR) further confirmed the successful formation of CeO_2_, with a prominent absorption peak at 593.01 cm^−1^, characteristic of Ce-O stretching vibrations within the lattice (Fig. 2F).

Previous studies have demonstrated that integrating CeO_2_ with mesenchymal stem cell (MSC)-derived vesicles markedly enhances both anti-inflammatory and antioxidant activities, offering considerable therapeutic potential for periodontitis treatment [52]. Zeta potential measurements revealed surface charges of +23.11 mV for CeO_2_ and -11.62 mV for EVs, enabling their facile assembly via electrostatic adsorption. Further optimized the mixing ratio yielded a CeO_2_-EV hybrid with a zeta potential of −5.41 mV (Fig. 2G and H). TEM and fluorescence microscopy co-localization confirmed that numerous CeO_2_ were densely distributed over the EVs surface, with the hybrid particles exhibiting a markedly increased diameter of approximately 110 nm compared with native EVs, indicative of the successful formation of a stable CeO_2_-EV nanohybrid system *in vitro* (Fig. 2I and J). There were no significant changes in the zeta potential and particle size of CeO_2_-EV in PBS and complete culture medium in 5 d. This result confirms the stability of the CeO_2_-EV (Fig. S4A and B). We next evaluated the therapeutic potential of the CeO_2_-EV composite, specifically its ability to scavenge H_2_O_2_ and ·O_2_^−^, two representative ROS. The total antioxidant capacity of EVs and CeO_2_ was first assessed using the FRAP assay, which is based on the reduction of ferric-tripyridyltriazine (Fe^3+^-TPTZ) to the blue Fe^2+^-TPTZ complex under acidic conditions. The yield of blue Fe^2+^-TPTZ is directly proportional to the total antioxidant capacity. The results showed that the optimal concentrations for EVs and CeO_2_ were determined to be 10 μg/mL and 50 μg/mL, respectively (Fig. 2K and L). These concentrations were then combined at a 1:1 vol ratio (5:1 mass ratio) to prepare a CeO_2_-EV composite suspension with a final concentration of 30 μg/mL. The enzymatic activities of CAT and SOD in the CeO_2_-EV composite were subsequently measured using a catalase assay kit and the WST-8 method, respectively. Compared to individual CeO_2_ and EVs, the CeO_2_-EV composite exhibited enhanced enzyme-like activity in simulating both CAT and SOD functions (Fig. 2M and N). The FRAP assay further confirmed that the CeO_2_-EV composite displayed superior total antioxidant capacity (Fig. 2O). These findings suggest that CeO_2_-EV hold significant potential for applications in antioxidant therapy and immune modulation.

### 3.3 In Vitro Antibacterial Activity of Ce-N-C@HA

Given the capability of the Ce-N-C@HA composite to catalyze the conversion of H_2_O_2_ into ·OH, we systematically investigated its antibacterial efficacy against *Staphylococcus aureus* (*S. aureus*), methicillin-resistant *Staphylococcus aureus* (MRSA), and *Porphyromonas gingivalis* (*P. gingivalis*). Firstly, we detected the growth kinetics of three bacterial strains by monitoring the OD 600 nm. *S. aureus* and MRSA achieved approximately 0.8 at 24 h, and *P. gingivalis* reached a comparable level at 48 h, indicating that these time points corresponded to the respective phases of optimal bacterial proliferation for subsequent antibacterial assays (Fig. S5A). To determine the optimum working concentration of Ce-N-C@HA that yields maximal antibacterial activity, 0.2 % H_2_O_2_ was employed as the reaction substrate, and three concentration gradients were tested. The results showed that all tested concentrations demonstrated measurable antibacterial activity. Notably, 100 μg/mL concentration achieving the most pronounced anti-bacterial effect so that we chose it as the subsequent working concentration (Fig. S5B).

**Fig. 3:**
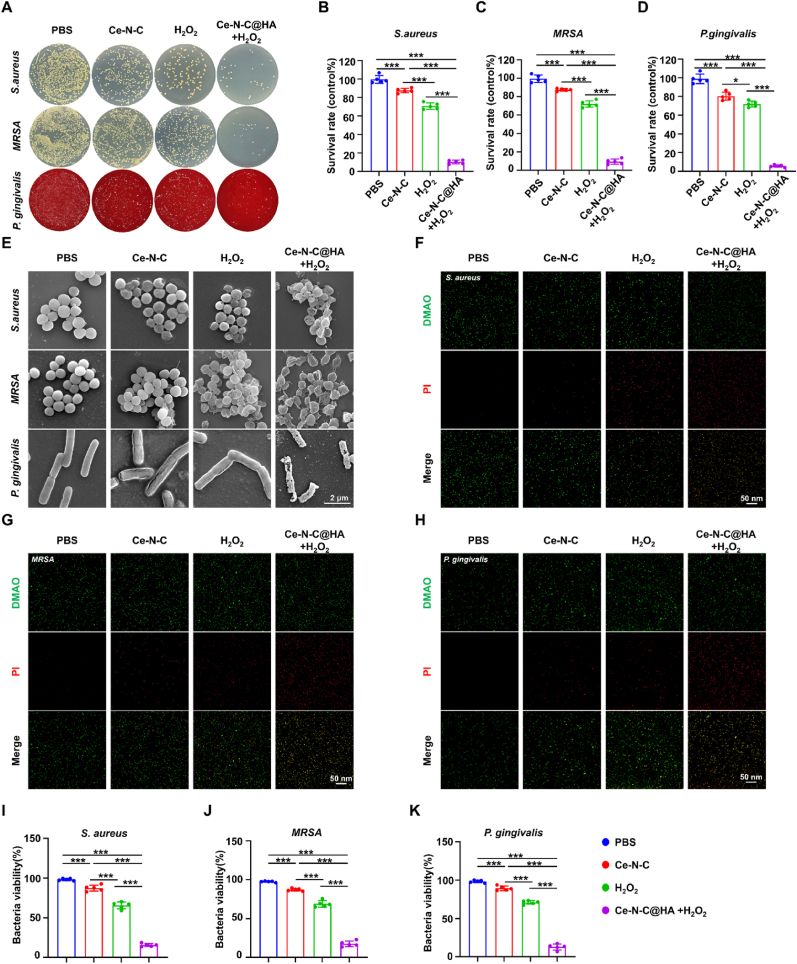
*In Vitro* Antibacterial Activity of Ce-N-C@HA. (A) Photographs of bacterial colonies formed by *S. aureus,* MRSA and *P. gingivalis*. (B–D) Quantification of the survival rate of *S. aureus,* MRSA and *P. gingivalis*. n = 5 per group. (E) Representative SEM images showing the morphology of *S. aureus,* MRSA and *P. gingivalis*. Bar: 2 μm. (F–H) Representative CLSM images for live/dead bacterial staining. Bar: 50 nm. (I–K) Quantification of bacteria viability. n = 5 per group. Data are presented as Mean ± SD. *∗P* < 0.05; *∗∗∗P* < 0.001.

To further elucidate the antibacterial mechanism of Ce-N-C@HA, we compared the bacterial manifestation following treatment under various conditions. In comparison to the PBS group, bacterial survival rates were notably reduced in all treatment groups (Fig. 3A). Specifically, in the Ce-N-C group, the survival rates in the three bacterial were 87.59 %, 87.20 %, and 81.38 %, suggesting that the Ce-N-C treatment alone had a limited antibacterial effect. Besides, the H_2_O_2_ group exhibited bacterial survival rates were 71.86 %, 70.11 %, and 67.84 %, indicating that H_2_O_2_ alone provided only a little antibacterial response, likely due to its relatively weak oxidative capacity. Notably, when Ce-N-C@HA was combined with H_2_O_2_, the antibacterial efficacy was markedly enhanced, with survival rates dropping to 9.61 %, 8.42 %, and 5.32 % for three bacterial, achieving a bactericidal efficiency exceeding 90 % (Fig. 3B–D). This enhanced effect can be attributed to Ce-N-C@HA catalyzing the decomposition of H_2_O_2_, resulting in the generation of highly reactive ·OH. Furthermore, SEM results revealed distinct morphological changes in the bacteria under different treatments. Bacteria in the PBS group exhibited smooth and intact surfaces, while only a minor fraction of bacteria in the Ce-N-C-treated group showed mild wrinkling, with the majority maintaining their structural integrity. Similarly, bacteria exposed solely to H_2_O_2_ displayed membrane wrinkling, reinforcing the notion that the antimicrobial activity of H_2_O_2_ alone is relatively weak. In contrast, when treated with both Ce-N-C@HA and H_2_O_2_, all three bacterial exhibited severe oxidative damage. This was evidenced by extensively wrinkled and ruptured membranes, the formation of irregular pores, and subsequent leakage of intracellular contents (Fig. 3E). These findings collectively demonstrate that the generated ·OH were shown to cause significant damage to the cell membranes of both Gram-positive and Gram-negative bacteria.

To quantitatively assess bacterial viability, we employed a DMAO/PI dual staining assay. DMAO, a nucleic acid-binding green fluorescent dye, readily penetrates bacterial membranes and binds to double-stranded DNA, generating a green fluorescence that marks both viable and dead bacteria. In contrast, propidium iodide can only enter bacteria with compromised membranes, where it binds to DNA and produces a red fluorescence, specifically labeling dead bacteria. Fluorescent staining results revealed that, in the PBS group, all three bacterial strains exhibited strong green fluorescence with minimal red fluorescence, indicating the predominance of viable bacteria. The Ce-N-C or H_2_O_2_ groups alone also displayed only partial red fluorescence, suggesting that most bacteria remained viable under these conditions. However, bacteria treated with Ce-N-C@HA in the presence of H_2_O_2_ exhibited intense red fluorescence, indicating a substantial increase in bacterial mortality (Fig. 3F–H). Quantitative analysis further confirmed these observations, revealing that the survival rates of the three bacterial strains in the Ce-N-C@HA-treated group decreased to 20.36 %, 15.23 %, and 12.04 %, respectively, with statistically significant differences compared to the control group (Fig. 3I–K). These results were in agreement with the colony count data, further supporting that Ce-N-C@HA exhibits remarkable peroxidase-like activity via the proportion of Ce^3+^ in the acidic microenvironment created by bacterial infection, effectively catalyzing the generation of highly ·OH from H_2_O_2_, thereby leading to direct damage of bacterial cell membranes and DNA as well as achieving potent bactericidal effects. In addition, the antibacterial performance of Ce NPs may be related to its surface morphology. The contact between sharp edges of Ce SAzymes and bacterial membranes could also affect bacterial activity.

### 3.4 In vitro anti-inflammatory function of CeO_2_-EV

To investigate the *in vitro* anti-inflammatory potential of CeO_2_-EV, immunofluorescence (IF) staining was first employed to examine cellular uptake by Raw264.7 cells. Distinct intracellular fluorescence signals were observed for CeO_2_-EV, indicating efficient cellular internalization and suggesting favorable membrane fusion that may facilitate subsequent biological activity (Fig. S6A and B). The biocompatibility of CeO_2_-EV was further evaluated through CCK-8 cytotoxicity assays and cell viability staining. CCK-8 results revealed that CeO_2_ at concentrations below 75 μg/mL did not inhibit cell proliferation, while EVs below 10 μg/mL displayed negligible cytotoxicity (Fig. S6C and D). Consistently, live/dead staining demonstrated that, after 24 h exposure to various concentrations of EVs, CeO_2_ and CeO_2_-EV, Raw264.7 cells viability in all groups remained above 80 %, confirming their favorable cytocompatibility (Fig. S6E–G). Taking into account the intrinsic antioxidant enzyme-mimicking activity of CeO_2_-EV, the optimal working formulation was determined to be a composite suspension prepared by mixing 50 μg/mL CeO_2_ with 10 μg/mL EVs (V_CeO2_/V_EVs_:1:1; M_CeO2_/M_EVs_:5:1), yielding a final CeO_2_-EV concentration of 30 μg/mL. This formulation ensured both biosafety and superior antioxidant performance.

**Fig. 4:**
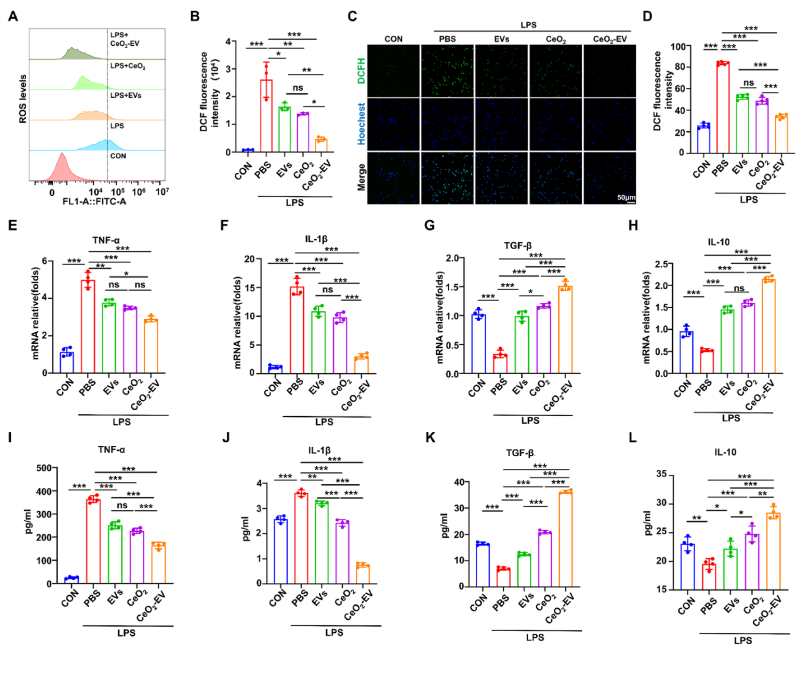
CeO_2_-EV relieved the oxidative stress state of activated macrophages. (A) FC histograms and (B) the quantification of ROS levels. n = 3 per group. (C) Representative DCFH-DA staining images and (D) the quantification of ROS expression level. Bar: 50 μm. n = 5 per group. (E–H) The cytokines gene expression levels of TNF-α, IL-1β, TGF-β and IL-10. n = 4 per group. (I–L) ELISA analysis of the cytokines of TNF-α, IL-1β, TGF-β and IL-10. n = 4 per group. Data are presented as Mean ± SD. *∗P* < 0.05; *∗∗P* < 0.01; *∗∗∗P* < 0.001; ns, no significance.

During the immune response, macrophages initially adopt a pro-inflammatory M1 phenotype to eliminate invading pathogens, subsequently transitioning to an anti-inflammatory M2 phenotype once danger signals subside, thereby facilitating tissue repair. This phenotypic shift is closely linked to intracellular ROS levels. Accordingly, we investigated the impact of CeO_2_-EV on ROS regulation in RAW264.7 cells. Given the potent antioxidant enzyme-mimicking activity of CeO_2_-EV, its regulatory effects on intracellular ROS were first examined. Flow cytometry (FC) results revealed a marked increase in ROS levels following LPS stimulation, whereas treatment with CeO_2_, EVs, or CeO_2_-EV significantly attenuated ROS accumulation, with the most pronounced reduction observed in the CeO_2_-EV group (Fig. 4A). Consistently, DCFH-DA fluorescence staining confirmed strong oxidative stress activation in LPS-treated cells, which was mitigated to varying degrees by all three treatments, with CeO_2_-EV again exhibiting the most substantial effect (Fig. 4B). Quantitative analysis further showed that CeO_2_-EV achieved a ROS scavenging rate of up to 80 % (Fig. 4C and D). Given the pivotal role of ROS in regulating macrophage cytokines secretion and polarization, we next assessed macrophage functional responses following treatment. Quantitative reverse transcription polymerase chain reaction (qRT-PCR) analysis demonstrated that all three treatments significantly down-regulated the mRNA expression of pro-inflammatory cytokines TNF-α and IL-1β in LPS-stimulated RAW264.7 cells, while up-regulating anti-inflammatory cytokines TGF-β and IL-10, with CeO_2_-EV displaying the most pronounced regulatory effect (Fig. 4E–H). Similarly, ELISA results corroborated these findings, showing that CeO_2_-EV markedly enhanced the secretion of TGF-β and IL-10 while suppressing TNF-α and IL-1β production (Fig. 4I–L). Collectively, these results indicate that CeO_2_-EV effectively mitigates oxidative stress in LPS-pretreated macrophages, thereby modulating the immune microenvironment toward a less inflammatory state.

**Fig. 5:**
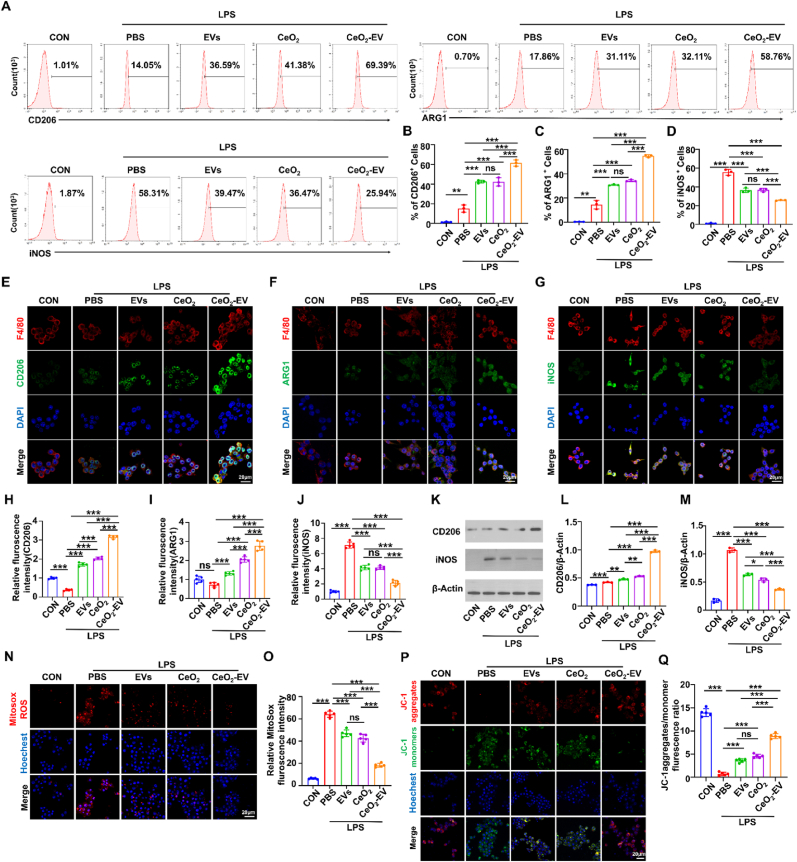
CeO_2_-EV regulates the polarization phenotype of macrophages and restores mitochondrial function. (A) FC and (B–D) the quantification of CD206, ARG1 and iNOS expression levels in RAW264.7 cells. n = 3 per group. (E–G) Representative IF staining and (H–J) the quantitative analysis of CD206, ARG1 and iNOS in RAW264.7 cells. Bar: 20 μm. n = 5 per group. (K) WB analysis of the RAW264.7 cells phenotypic marker expression. Relative gray value statistics of (L) CD206 and (M) iNOS. n = 3 per group. (N) Fluorescence staining of Mitochondrial ROS levels and (O) the relative quantitative analysis. Bar: 20 μm. n = 5 per group. (P) Fluorescence staining of the level of mitochondrial membrane potential change and (Q) the relative quantitative analysis. Bar: 20 μm. n = 5 per group. Data are presented as Mean ± SD. *∗P* < 0.05; *∗∗P* < 0.01; *∗∗∗P* < 0.001; ns, no significance.

To further verify the ability of CeO_2_-EV to induce an anti-inflammatory phenotype in RAW264.7 macrophages, phenotypic marker analysis was conducted following treatment. FC results revealed that LPS stimulation markedly suppressed the expression of M2 associated markers CD206 and ARG1, while strongly up-regulating the M1-associated marker iNOS. In contrast, treatment with EVs, CeO_2_ or CeO_2_-EV effectively restored CD206 and ARG1 expression and concomitantly suppressed iNOS levels, with CeO_2_-EV exerting the most pronounced regulatory effects. Specifically, the proportions of CD206 positive and ARG1 positive cells in the CeO_2_-EV group reached 69.39 % and 58.76 %, respectively, whereas the proportion of iNOS positive cells decreased to 25.94 % (Fig. 5A–D). IF staining further confirmed these findings, showing robust expression of CD206 and ARG1 and minimal iNOS expression in the CeO_2_-EV group (Fig. 5E–J). Consistent with these results, Western blot (WB) analysis demonstrated that LPS stimulation significantly increased iNOS and reduced CD206 protein levels, whereas CeO_2_-EV treatment reversed these changes, markedly down-regulating iNOS and up-regulating CD206 expression (Fig. 5K–M). In our design, CeO_2_ was conjugated to EVs surface, which enables EVs to play a role in both as delivery vehicles and intrinsic anti-inflammatory agents. This ROS-scavenging, combined strategy enhances CeO_2_ antioxidant and anti-inflammatory potency. Importantly, dissociated CeO_2_ and EVs entered the cell and retained independent therapeutic activity (Fig. S6A and B), yet CeO_2_-EV can also act cooperatively to achieve amplified anti-inflammatory effects. Collectively, these data demonstrate that CeO_2_-EV possesses potent immunomodulatory activity, suppressing excessive inflammatory responses in macrophages and promoting their polarization toward the M2 phenotype.

To gain deeper insights into the molecular mechanisms by which CeO_2_-EV modulate RAW264.7 cell functions, we performed RNA sequencing (RNA-seq) on LPS-pretreated RAW264.7 cells following CeO_2_-EV treatment (Fig. S7A). A total of 211 DEGs were identified, including 103 up-regulated and 108 down-regulated genes (Fig. S7B). GO enrichment analysis revealed that CeO_2_-EV treatment significantly affected the inflammation-related genes and signaling pathways expression. This included well-established pathways involved in macrophage programming, such as the NF-κB, MAPK, and JAK-STAT signaling pathways, which are known to promote M1-like macrophage polarization (Fig. S7C-E). Furthermore, KEGG analysis indicated a marked up-regulation of anti-inflammatory-related genes (Fig. S7F), supporting the strong immunomodulatory capacity of CeO_2_-EV.

Macrophage polarization is deeply influenced by mitochondrial function and structural integrity [53]. The transition to pro-inflammatory, M1-like phenotypes is mainly driven by mitochondrial dysfunction, especially the loss of membrane potential and excessive ROS. Given the pivotal role of mitochondria as the cellular powerhouse [54], we next examined mitochondrial functional changes during CeO_2_-EV mediated phenotypic conversion of RAW264.7 cells. MitoSOX Red staining was first employed to quantify mitochondrial ROS levels. LPS stimulation induced a marked elevation in mitochondrial ROS, indicative of mitochondrial dysfunction; however, treatment with EVs, CeO_2_, or CeO_2_-EV markedly attenuated this effect, with CeO_2_-EV restoring mitochondrial ROS levels to near those of the control group (Fig. 5N and O). Subsequently, mitochondrial membrane potential was assessed using the JC-1 fluorescent probe. In LPS-treated RAW264.7 cells, ROS accumulation initiated a mitochondrial damage cascade, disrupting the electron transport chain and resulting in dominant JC-1 monomer (Green) fluorescence with a near-complete loss of JC-1 aggregate (Red) signal, indicative of membrane potential collapse. In contrast, all treatment groups exhibited increased JC-1 aggregate/monomer ratios, reflecting recovery of mitochondrial membrane potential, with CeO_2_-EV producing the most pronounced restoration and showing no significant difference from the control group (Fig. 5P and Q). To further explore the relationship between M2 polarization and the restoration of mitochondrial function, we conducted a more detailed analysis of the RNA-seq data. Up-regulated DEGs were annotated into three major GO categories. Notably, in both the “Biological Process” and “Molecular Function” categories, there was significant enrichment of DEGs associated with enhanced mitochondrial function (Fig. S8A and B). These included pathways such as “Mitochondrial electron transport, ubiquinol to cytochrome *c*,” “Energy derivation by oxidation of organic compounds,” “Calcium signaling pathway,” and “PPAR signaling pathway” (Fig. S8C). These results confirmed that CeO_2_-EV treatment significantly enhances mitochondrial function in RAW264.7 cells, which were consistent with our fluorescence staining data. Together, these findings support a close association between the recovery of mitochondrial function and M2 polarization. Collectively, these findings demonstrate that CeO_2_-EV not only scavenges ROS to modulate the inflammatory cytokines profile and drive macrophage polarization toward the M2 phenotype, but also repairs mitochondrial dysfunction, thereby suppressing excessive immune-inflammatory responses.

### 3.5 In vivo antibacterial efficacy of Ce-N-C@HA for the improvement of periodontitis

**Fig. 6:**
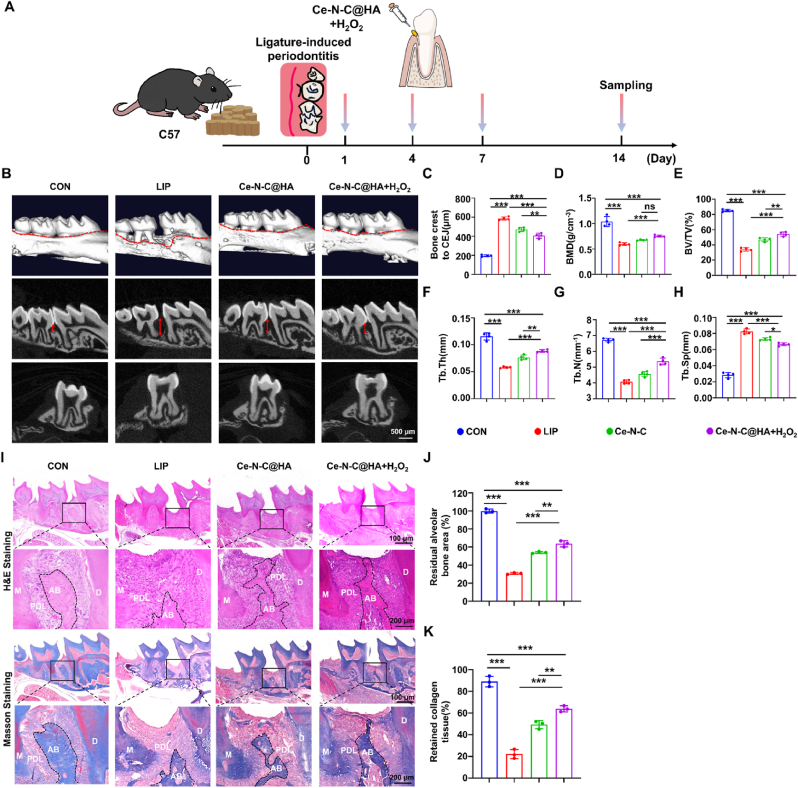
The antibacterial efficacy of Ce-N-C@HA for the improvement of periodontitis *in vivo*. (A) Schematic diagram for the Ce-N-C@HA treatment procedure of periodontitis. (B) Representative images of the CBCT-based three-dimensional reconstruction (Red line: bone loss; Red arrow: Bone crest to CEJ). Bar: 500 μm. (C) Quantification analysis of the bone crest to CEJ distance. (D) The quantification of the BMD, (E) BV/TV, (F) Tb. Th, (G) Tb. N and (H) Tb. Sp calculated from CBCT reconstructed images. n = 4 per group. (I) Representative images of the H&E/Masson staining images of the periodontitis. Bar: 100/200 μm (M: Mesial surface; D: Distal surface; PDL: periodontal ligament; AB: alveolar bone). (J) Residual alveolar bone area percentage and (K) retained collagen tissue percentage of Masson staining. n = 3 per group. Data are presented as Mean ± SD. *∗P* < 0.05; *∗∗P* < 0.01; *∗∗∗P* < 0.001; ns, no significance. (For interpretation of the references to colour in this figure legend, the reader is referred to the Web version of this article.)

The periodontal microenvironment is inherently complex, with pathological periodontal pockets harboring abundant pathogenic microorganisms that secrete enzymes, toxins, and metabolic byproducts. These deleterious factors disrupt periodontal tissue integrity, provoke persistent inflammatory responses, and ultimately result in irreversible damage, including alveolar bone resorption and loss of attachment. Therefore, the prompt eradication of pathogenic bacteria is essential for effective periodontitis management. Building on the potent *in vitro* antibacterial properties of Ce-N-C materials, we employed a murine periodontitis model to investigate the bactericidal activity of Ce-N-C@HA against bacterial biofilms within periodontal pockets and to evaluate its therapeutic efficacy *in vivo* (Fig. 6A). CBCT-based three-dimensional reconstruction revealed that, relative to the CON group (194.75 μm), the LIP group (586.50 μm) exhibited pronounced alveolar bone resorption, as evidenced by a marked increase in the bone crest to cementoenamel junction (CEJ) distance, thereby confirming successful model establishment. In contrast, treatment with Ce-N-C@HA (472.75 μm) and Ce-N-C@HA + H_2_O_2_ (406.75 μm) effectively reduced the bone crest to CEJ distance, with the latter demonstrating a more substantial inhibition of bone resorption (Fig. 6B and C). Quantitative assessment of bone microarchitectural parameters showed that the bone mineral density (BMD, 0.59 g/cm^3^) and bone volume fraction (BV/TV, 34 %) were significantly decreased in the LIP group, indicating a substantial loss of alveolar bone mass and density following LPS-induced inflammation. Conversely, Ce-N-C@HA (BMD, 0.67 g/cm^3^; BV/TV, 46.92 %) and Ce-N-C@HA + H_2_O_2_ (BMD, 0.74 g/cm^3^; BV/TV, 54.33 %) treatments markedly restored both parameters, achieving statistically significant improvements compared with the LIP group. Notably, Ce-N-C@HA + H_2_O_2_ also elicited more pronounced trabecular bone regeneration, characterized by increased trabecular number (Tb. N) and thickness (Tb. Th) alongside a reduction in trabecular separation (Tb. Sp) (Fig. 6D–H).

Additionally, to further substantiate the anti-resorptive potential of the composite material, histological evaluation was conducted on alveolar bone subjected to different treatment conditions. Hematoxylin-Eosin (H&E) staining revealed that, relative to the LIP group, the Ce-N-C@HA + H_2_O_2_ group exhibited a marked restoration of alveolar bone morphology and area, with the recovered area reaching 63.73 %. Masson staining further demonstrated that, in the LIP group, blue-stained collagen tissues appeared disorganized and were interspersed with red-stained inflammatory regions, indicative of collagen degradation or even rupture, particularly along the alveolar bone margins and at periodontal ligament attachment sites. In contrast, following Ce-N-C@HA + H_2_O_2_ treatment, the periodontal collagen tissues were notably restored, with fiber density reaching 63.96 % (Fig. 6I–K). It is noteworthy that although *Porphyromonas gingivalis* lacks hyaluronidase activity [5], various bacteria in the oral microenvironment can secrete hyaluronidase**.** These findings confirm that, under *in vivo* conditions, Ce-N-C@HA can be degraded by bacterial hyaluronidase, thereby catalyzing the conversion of H_2_O_2_ into elevated levels of reactive ·OH, effectively eradicating pathogenic bacterial biofilms within periodontal pockets and facilitating the subsequent repair of periodontal tissues.

### 3.6 Immune microenvironment modulation by CeO_2_-EV to treat periodontitis

**Fig. 7:**
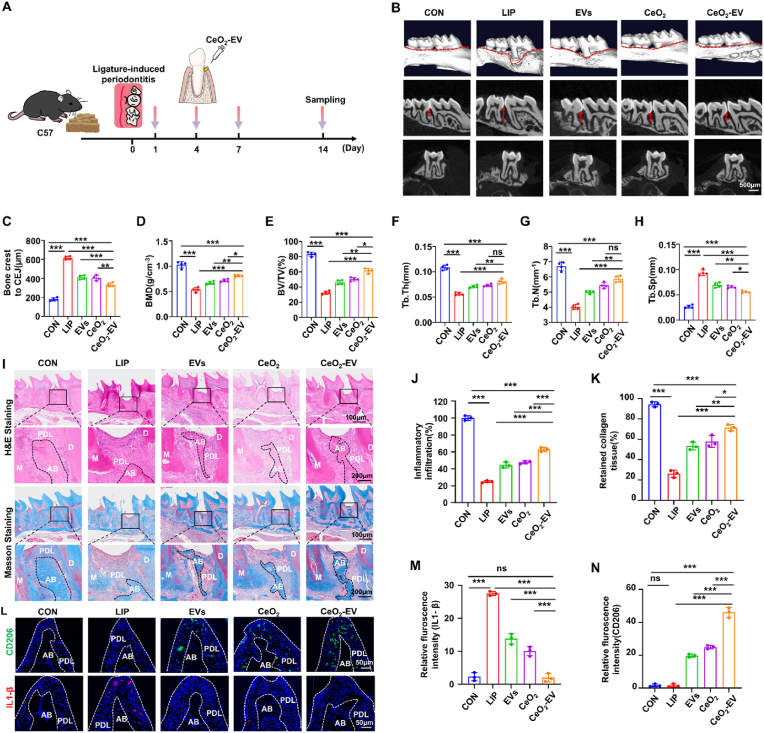
CeO_2_-EV modulates the immune microenvironment to improve periodontitis *in vivo*. (A) Schematic diagram for the CeO_2_-EV treatment procedure of periodontitis. (B) Representative images of the CBCT-based three-dimensional reconstruction. Bar: 500 μm. (C) The quantification analysis for the bone crest to CEJ distance. (D) The quantification of the BMD, (E) BV/TV, (F) Tb. Th, (G) Tb. N and (H) Tb. Sp calculated from CBCT reconstructed images. n = 4 per group. (I) Representative images of the H&E/Masson staining images of the periodontitis. Bar: 100/200 μm. (J) Residual alveolar bone area percentage and (K) retained collagen tissue percentage of Masson staining. n = 3 per group. (L) Representative IF images of CD206 (Green) and IL-1β (Red) in the alveolar bone region and (M − N) relative fluorescence intensities. Bar: 50 μm. n = 3 per group. Data are presented as Mean ± SD. *∗P* < 0.05; *∗∗P* < 0.01; *∗∗∗P* < 0.001; ns, no significance. (For interpretation of the references to colour in this figure legend, the reader is referred to the Web version of this article.)

The excessive activation of macrophages constitutes one of the earliest inflammatory events following periodontal injury. In the initial stages, macrophages accumulate at the inflammatory site and undergo oxidative stress, leading to a pronounced imbalance in polarization, predominantly skewing toward the pro-inflammatory M1 phenotype. These M1 phenotype macrophages secrete a range of pro-inflammatory cytokines that further recruit diverse inflammatory cell populations, amplifying and sustaining an uncontrolled inflammatory cascade. Consequently, modulating macrophage polarization from the M1 phenotype toward the M2 phenotype in periodontitis represents a promising strategy to suppress excessive inflammatory responses. To investigate this therapeutic approach, a periodontitis model was established in the maxillary second molars of mice via silk ligation, and the *in vivo* efficacy of the CeO_2_-EV composite was subsequently evaluated (Fig. 7A). CBCT-based three-dimensional reconstruction revealed that the LIP group exhibited marked alveolar bone resorption and gingival recession, with distinct areas of bone loss highlighted in yellow. In contrast, the EVs, CeO_2_, and CeO_2_-EV treated groups demonstrated a substantial reduction in bone resorption areas (Fig. 7B). Quantitative measurements indicated that the mean distance from the bone crest to CEJ in the LIP group (615.75 μm) was significantly increased than in the CON group (180.00 μm). Treatment with EVs (411.00 μm), CeO_2_ (404.25 μm), or CeO_2_-EV (344.25 μm) notably reduced this distance, with the CeO_2_-EV group achieving the most pronounced improvement in bone preservation (Fig. 7C). Consistently, microstructural bone parameters demonstrated that the CeO_2_-EV group exhibited markedly higher bone mineral density (BMD, 0.81 g/cm^3^, BV/TV, 61.37 %) compared with the EVs group (BMD, 0.67 g/cm^3^; BV/TV, 46.33 %) and the CeO_2_ group (BMD, 0.71 g/cm^3^; BV/TV, 50.30 %). Moreover, Tb. N and Tb. Th were significantly elevated in all treatment groups relative to the LIP group, while Tb. Sp was significantly reduced, with the CeO_2_-EV group again showing the most substantial recovery in microarchitectural integrity (Fig. 7D–H). Furthermore, H&E staining revealed that periodontal tissues in the LIP group displayed extensive infiltration of inflammatory cells accompanied by pronounced loss of alveolar bone structure. Following intervention with EVs, CeO_2_, or CeO_2_-EV, the extent of inflammatory cell infiltration was markedly attenuated, and the morphology of the alveolar bone showed varying degrees of restoration, with the CeO_2_-EV group exhibiting the most pronounced regenerative effect. Masson staining further demonstrated that, relative to the other treatment groups, CeO_2_-EV administration significantly mitigated collagen degradation within periodontal tissues, as evidenced by a higher collagen density and a more orderly fiber arrangement (Fig. 7I–K). Collectively, these findings confirm that EVs, CeO_2_, and particularly the CeO_2_-EV, confer significant therapeutic benefits in the treatment of experimental periodontitis in mice, with the CeO_2_-EV group achieving superior histological and structural recovery.

During the tissue regeneration phase, macrophages recruited to the inflammatory site and secrete multiple growth factors for modulating collagen and fiber synthesis, thereby playing a pivotal role in the reparative process [55]. Accordingly, we investigated alterations in macrophage phenotype. IF staining revealed that local administration of EVs, CeO_2_, or CeO_2_-EV markedly increased the proportion of CD206 positive cells within the newly formed tissue in all treatment groups, with the most pronounced elevation observed in the CeO_2_-EV group. Conversely, the proportion of IL-1β positive cells was substantially elevated in the LIP group, reflecting a sustained inflammatory response. In contrast, IL-1β expression was reduced in all treatment groups, with the CeO_2_-EV group exhibiting the most significant decrease, in which the average expression level declined to 7.17 % (Fig. 7L–N). Collectively, these results demonstrate that local delivery of CeO_2_-EV effectively drives macrophage polarization toward the M2 phenotype, mitigates inflammatory activity in the periodontal tissue microenvironment, and consequently enhances alveolar bone repair and collagen regeneration, ultimately facilitating functional recovery of periodontal tissue.

### 3.7 Combined effect of a dual Ce-based nano-system

**Fig. 8:**
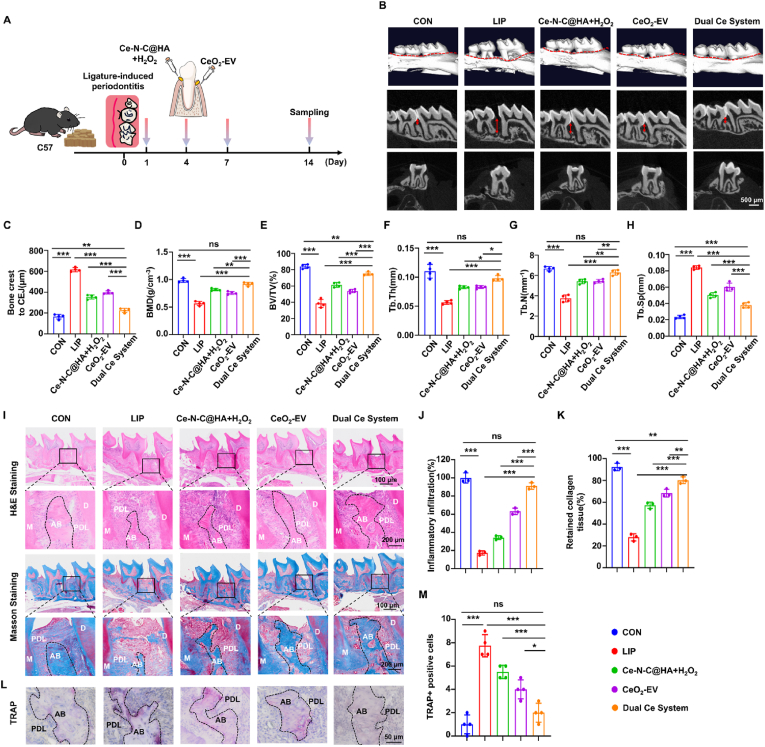
Dual Ce system reduced alveolar bone loss in mouse models of periodontitis. (A) Schematic diagram for the Dual Ce system treatment procedure of periodontitis *in vivo*. (B) Representative images of the CBCT-based three-dimensional reconstruction. Bar: 500 μm. (C) The quantification analysis for the bone crest to CEJ distance. (D) The quantification of the BMD, (E) BV/TV, (F) Tb. Th, (G) Tb. N and (H) Tb. Sp calculated from CBCT reconstructed images. n = 4 per group. (I) Representative images of the H&E/Masson staining images of the periodontitis. Bar: 100/200 μm. (J) Residual alveolar bone area percentage and (K) retained collagen tissue percentage of Masson staining. n = 3 per group. (L) Representative Trap staining images and (M) the quantification of Trap positive cells in the alveolar bone region. Bar: 50 μm. n = 4 per group. Data are presented as Mean ± SD. *∗P* < 0.05; *∗∗P* < 0.01; *∗∗∗P* < 0.001; ns, no significance.

Preliminary experiments indicated that intra-pocket administration of Ce-N-C@HA + H_2_O_2_ effectively targeted bacterial biofilms, achieving potent bactericidal effects, whereas subgingival tissues injection of CeO_2_-EV markedly modulated the immune microenvironment. Based on these findings, a dual-module cerium-based nanosystem was constructed to achieve modulation of the periodontal microenvironment through the combined bactericidal and immunoregulatory actions, thereby facilitating periodontal tissue regeneration. Following 14d of treatment, samples from each group were collected for comprehensive evaluation (Fig. 8A). CBCT-based three-dimensional reconstructions revealed pronounced alveolar bone loss in the LIP group (Fig. 8B). Quantitative measurements demonstrated that the mean distance from the bone crest to the CEJ in the Ce-N-C@HA + H_2_O_2_ group (354.00 μm) and the CeO_2_-EV group (396.80 μm) was markedly reduced compared with the LIP group (616.50 μm), yet remained significantly greater than that of the control group (165.50 μm), indicating that monotherapy with either module provides only partial bone repair. Strikingly, the Dual Ce System group (225.75 μm) exhibited substantial bone regeneration and approaching the physiological level observed in controls (Fig. 8C). Furthermore, BMD and BV/TV within the interproximal region between the first and second molars were significantly increased in the Dual Ce System group. Microarchitectural analyses revealed that Tb. N and Tb. Th were higher, while Tb. Sp was reduced, relative to both single-module treatment groups (Fig. 8D–H). Collectively, these results consistently demonstrate that monotherapy with cerium-based nanomaterials yields limited bone repair, whereas the Dual Ce System achieves superior regenerative outcomes in periodontitis. In addition, H&E and Masson staining revealed that, relative to the LIP group, both Ce-N-C@HA + H_2_O_2_ group and CeO_2_-EV group mitigated alveolar bone destruction and attenuated collagen fiber degradation; however, the extent of tissue regeneration in the Dual Ce System group was most comparable to that of the CON group. This was characterized by a pronounced reduction in inflammatory cell infiltration, re-establishment of an intact gingival epithelial layer and connective tissue architecture, formation of a dense and uniformly aligned collagen fiber network, and a substantial increase in alveolar bone area (Fig. 8I–K). Tartrate-resistant acid phosphatase (TRAP) staining results further indicated that the Dual Ce System markedly suppressed osteoclast activity induced by periodontitis, thereby effectively reducing alveolar bone resorption (Fig. 8L–M). In addition, *in vivo* imaging was used to detect the retention of materials in periodontal tissues. CeO_2_-EV and Ce-N-C@HA were respectively injected into the periodontal pockets and tissue epithelium of mice. The results demonstrated that CeO_2_-EV and Ce-N-C@HA were predominantly localized to the periodontal site on 0 h. Their total flux[p/s] were reached its peak at 0h post-injection. With the prolongation of time, the signals gradually diminished over the next two days and were completely cleared by 72 h. Both of the total flux [p/s] were nearly undetectable by 72 h (Fig. S9A and B). These histological observations, together with the imaging analyses, consistently highlight the superior regenerative efficacy of the Dual Ce System in restoring periodontal tissue integrity.

**Fig. 9:**
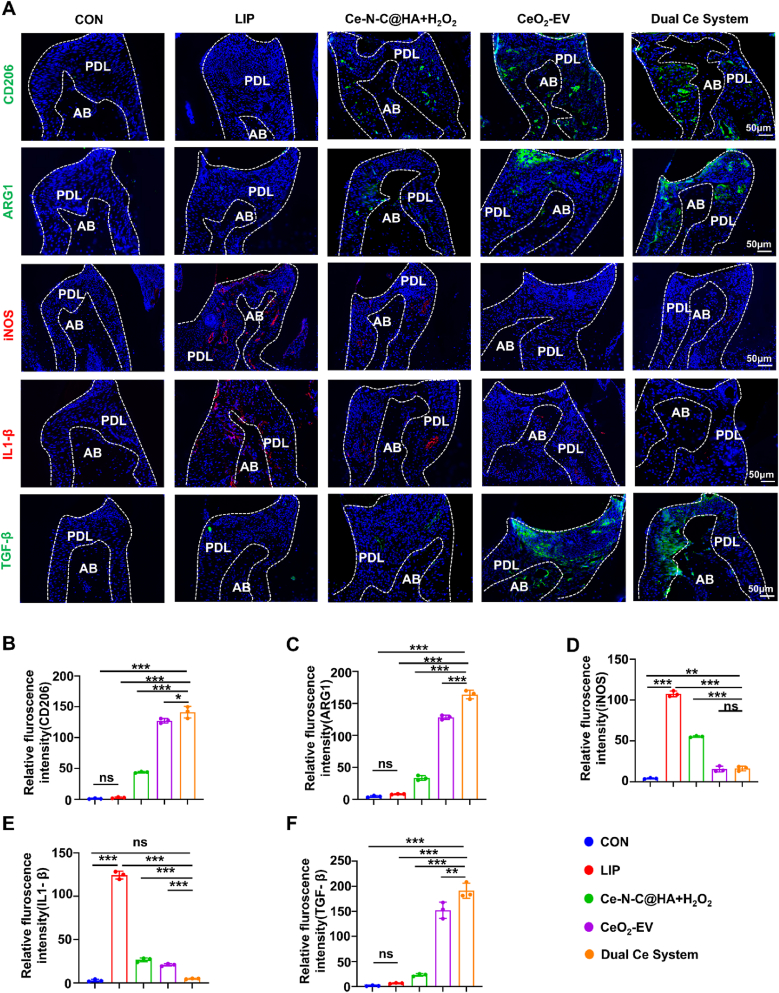
Dual Ce system relieved periodontal tissue inflammation. (A) Representative IF staining images of CD206, ARG1, iNOS and IL-1β and TNF-α in the alveolar bone region. Bar: 50 μm. (B) The quantification analysis of CD206, (C) ARG1, (D) iNOS, (E) IL-1β and (F) TNF-α. n = 3 per group. Data are presented as Mean ± SD. *∗P* < 0.05; *∗∗P* < 0.01; *∗∗∗P* < 0.001; ns, no significance.

Additionally, analysis of the immune microenvironment provided further insight into the underlying mechanism of the combined action exerted by the Dual Ce System. IF staining revealed that the fluorescence intensities of CD206 and ARG1 in the Dual Ce System group were markedly elevated compared with those in the LIP group, representing the highest levels among all treatment groups. In contrast, the fluorescence intensity of iNOS was significantly reduced relative to the LIP group, indicating that the Dual Ce System effectively facilitated the polarization of RAW264.7 cells toward the M2 phenotype while suppressing their shift toward the M1 phenotype. Consistent with these findings, further assessment of intracellular inflammation-related factors demonstrated that all treatment groups significantly down-regulated the expression of IL-1β, with average fluorescence intensities decreasing to 21.45 % in the Ce-N-C@HA + H_2_O_2_ group, 16.82 % in the CeO_2_-EV group, and a notably low 3.92 % in the Dual Ce System group. Strikingly, the Dual Ce System group also induced a pronounced up-regulation of the anti-inflammatory cytokine TGF-β, compared to the LIP group (Fig. 9A–F). Moreover, gingival crevicular fluid samples collected from mice were subjected to smear examination followed by strict anaerobic cultivation for 72 h, revealing that both the Ce-N-C@HA + H_2_O_2_ group and the Dual Ce System group exhibited a substantial reduction in the abundance of *Porphyromonas gingivalis* compared with the LIP group (Fig. S10). Collectively, these multidimensional results underscore that the Dual Ce System achieves its combined enhancement via a modular design: Ce-N-C@HA + H_2_O_2_, localized within the periodontal pocket, exerts potent antibacterial effects, whereas CeO_2_-EV, delivered into the subgingival tissues, drives immune modulation. This dual-module strategy not only alleviates key pathological processes of periodontitis such as inhibiting bone resorption and mitigating collagen degradation-but also efficiently promotes bone and collagen repair, ultimately demonstrating remarkable overall therapeutic efficacy against periodontitis.

### 3.8 Biosafety of the dual Ce system

To comprehensively evaluate the *in vivo* safety of the Dual Ce System, we systematically analyzed the body weight profiles and histopathological features of major internal organs following administration. Specifically, C57BL/6 mice were intravenously injected with the Ce-N-C@HA and CeO_2_-EV composite formulation via the tail vein, and body weight was continuously monitored over a 6 d period. The growth curves of the Dual Ce System group closely overlapped with those of the CON group, showing no statistically significant differences (Fig. S11A), thereby indicating that the treatment did not interfere with normal physiological development. Subsequently, co-administration of Ce-N-C@HA and CeO_2_-EV was performed via tail vein injection, and after 2 w, major organs including the heart, liver, spleen, lung, and kidney were harvested for histopathological evaluation. H&E staining revealed well-preserved tissue architecture in all organs of the Dual Ce System group, with no evident inflammatory infiltration, necrosis, or structural abnormalities, consistent with the morphological features observed in the CON group (Fig. S11B). Furthermore, serum biochemical analyses demonstrated that all measured parameters in the Dual Ce System group were within physiological ranges and statistically indistinguishable from those of the CON group (Fig. S11C–I), suggesting the absence of hepatic or renal functional impairment. Collectively, these findings confirm that the Dual Ce System possesses favorable biocompatibility under *in vivo* exposure conditions. This robust safety profile establishes a solid foundation for its potential clinical application and translational development.

## 4 Conclusion

This study pioneered a dual Ce-based nanozyme system, leveraging two structurally and functionally distinct cerium nanozymes composite EVs for antibacterial and anti-inflammatory periodontitis treatment. The structural discrepancy of Ce-N-C and CeO_2_ leads to different catalytic characteristics. The Dual Ce System consists of two therapeutic modules. The antibacterial module Ce-N-C@HA, which can be injected into pathological periodontal pockets, generates ·OH via catalytic reactions, effectively inhibiting the growth of *P. gingivalis*, thus combating periodontitis progression. The immune modulation module CeO_2_-EV demonstrates potent anti-inflammatory properties. It effectively scavenges ROS, promotes M2 phenotype polarization, restores mitochondrial function, and facilitates periodontal tissue recovery. In conclusion, the Dual Ce System elicits antibacterial and immunoregulatory dual effects via regulate ROS in tissue microenvironment, demonstrating robust therapeutic efficacy in periodontitis treatment. This study confirmed that cerium-based nanozymes with unique structural and functional characteristics have promising application prospects in the biomedical field.

Although cerium-based nanomaterials have achieved substantial advancements, clinical applications remain numerous challenges [56]. High temperature, high pressure and other preparation conditions impose significant constraints on the scale-up of the synthesis process. Owing to their elevated surface free energy, single metal atomic sites tend to undergo aggregation into nanoclusters, representing the primary bottleneck encountered during the fabrication process [57]. In the future, it remains to be studied to control the coordination number of individual metal atom sites to enhance the catalytic activity of SAzymes [58]. Furthermore, the prior to clinical implementation, critical questions concerning long-term biocompatibility profiles and *in vivo* metabolic pathways therefore necessitate rigorous investigation. The biological properties presented by Ce SAzymes still require in-depth mechanism research.

## CRediT authorship contribution statement

**Haozhe Ren:** Writing – original draft, Project administration, Investigation. **Peisheng Liu:** Conceptualization. **Ziang Sun:** Software. **Zhe Yu:** Resources. **Hao Guo:** Formal analysis. **Xinyue Cai:** Investigation. **Yihang Wei:** Methodology. **Zihan Li:** Formal analysis. **Meiling Wu:** Software. **Xinyue Xu:** Writing – review & editing. **Jing Wang:** Writing – review & editing. **Kun Xuan:** Writing – review & editing.

## Declaration of competing interest

The authors declare that they have no known competing financial interests or personal relationships that could have appeared to influence the work reported in this paper.

## Data Availability

Data will be made available on request.
