# Supplementary material for: Combined strategy of dual-module cerium nanosystem composite extracellular vesicles regulate ROS in the tissue microenvironment to promote periodontitis recovery
Source: Mater Today Bio. 2025 Dec 4;36:102625. doi: 10.1016/j.mtbio.2025.102625 (PMC12756039; doi:10.1016/j.mtbio.2025.102625)
Supplement: Multimedia component 1 [file mmc1.docx]

**Supplementary Figures and Figure Legends**


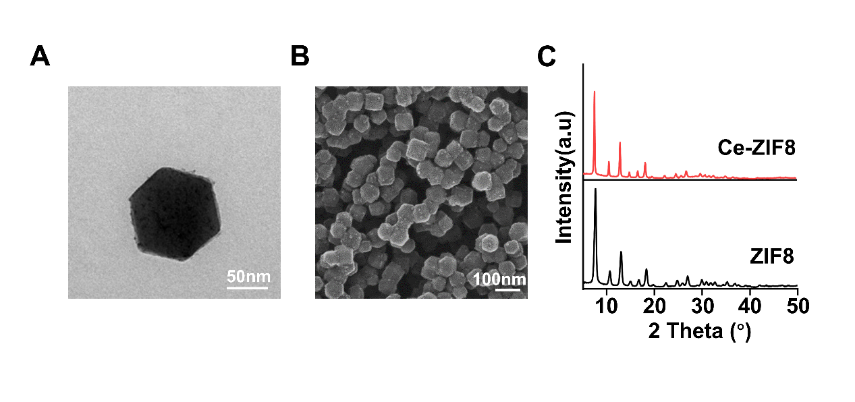


**Fig. S1.** Characterization of Ce-ZIF-8. (A) Representative TEM image showing the morphology of Ce-ZIF-8. Bar: 50 nm. (B) Representative SEM image showing the morphology of Ce-ZIF-8. Bar: 100 nm. (C) XRD images of Ce-ZIF-8 and ZIF-8.


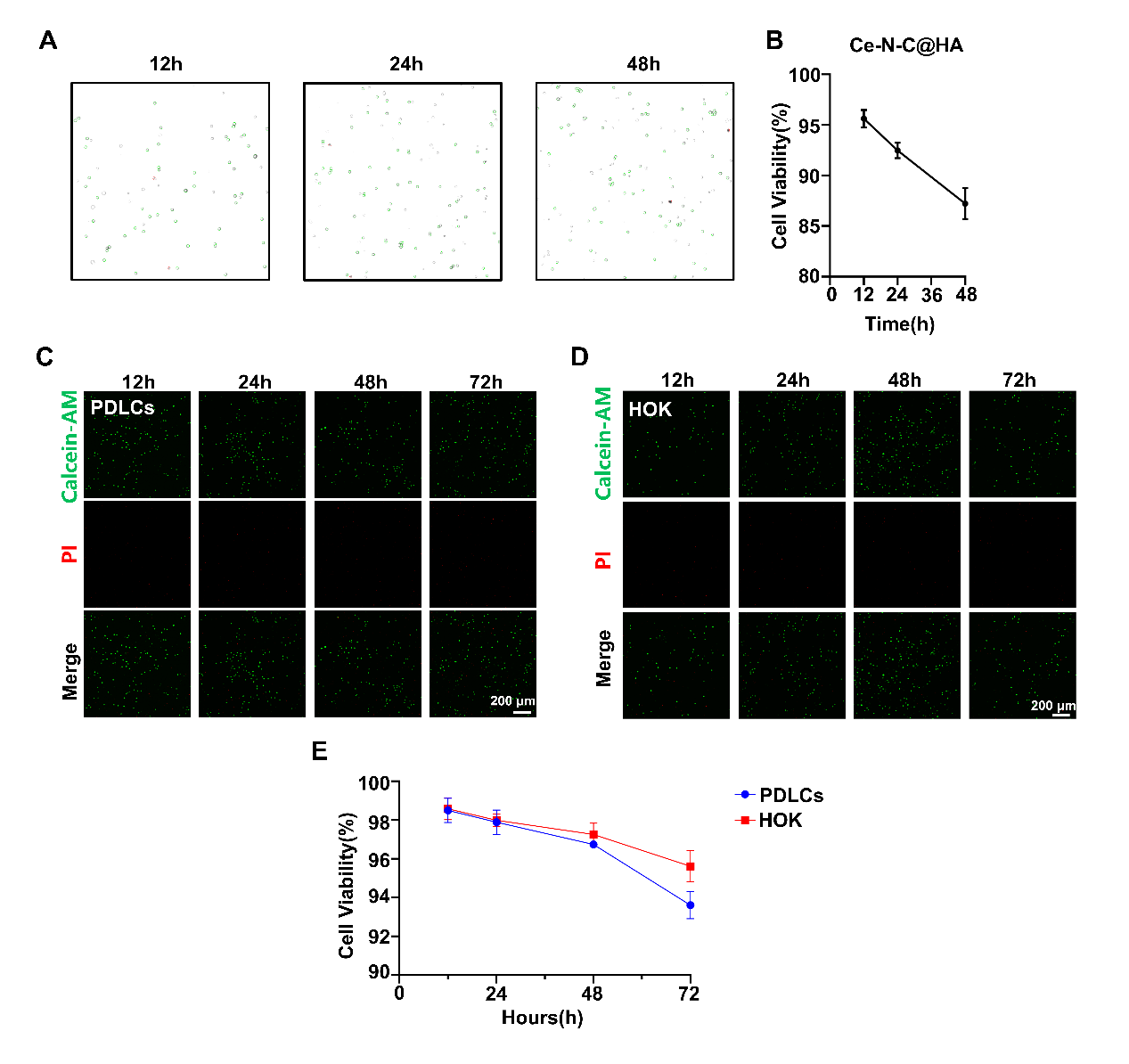


**Fig. S2.** Trypan blue staining results of Raw264.7 cells treated with Ce-N-C@HA. (A) Representative light microscopy images of trypan blue staining and (B) the quantification of cell viability percentage. n = 3 per group. (C-E) PDLCs and HOK cells viability of Ce-N-C@HA at varying concentrations using Live/Dead staining. Bar: 200 μm. Data are presented as Mean ± SD.


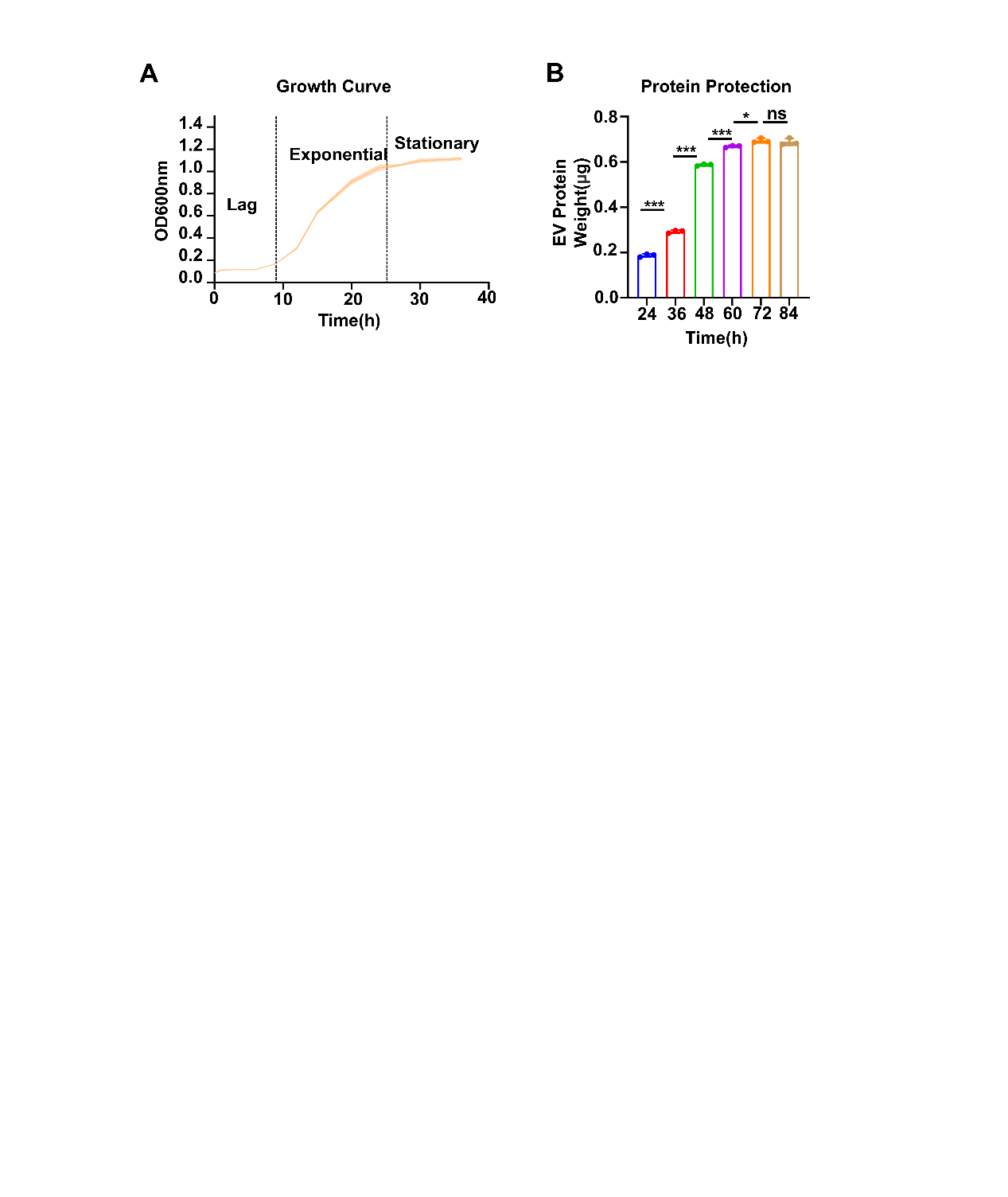


**Fig. S3.** Protein concentration assay for EVs. (A) Growth curve of *L. reuteri*. (B) BCA protein quantification for the protein production of EVs. n = 3 per group. Data are presented as Mean ± SD. **P* < 0.05; ****P* < 0.001; ns, no significance.


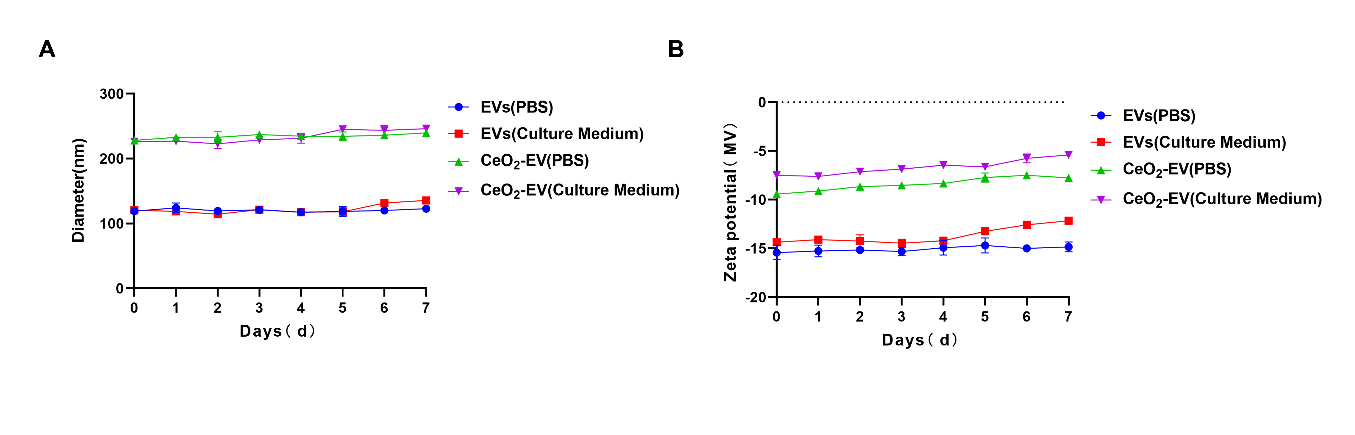


**Fig. S4.** The stability testing of CeO_2_-EV. (A) DLS and (B) ZETA potential measurements in PBS and culture medium. Data are presented as Mean ± SD.


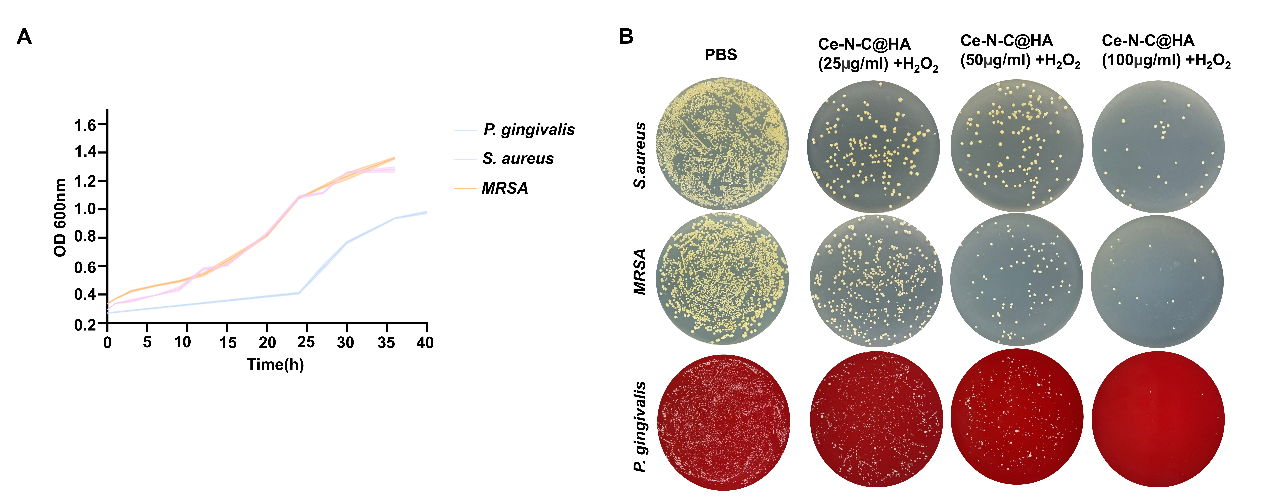


**Fig. S5.** The effective antibacterial concentration of Ce-N-C@HA. (A) Growth curve of *S. aureus*, MRSA, and *P. gingivalis*. (B) Antibacterial activity assay of Ce-N-C@HA at different concentrations.


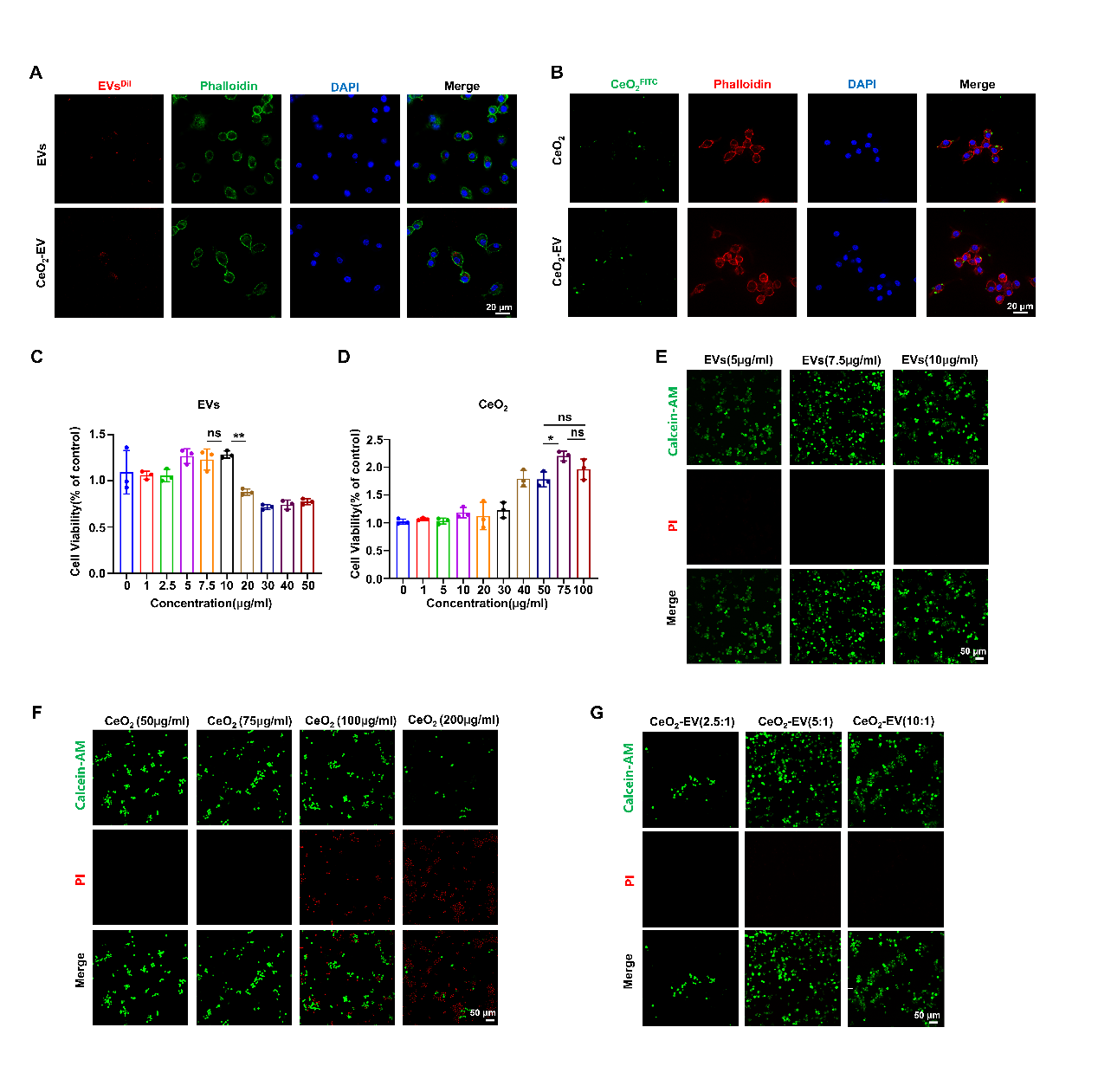


**Fig. S6.** The cytocompatibility of CeO_2_-EV. (A) Fluorescence of EVs (Dil, red) and F-actin cytoskeleton (Phalloidin, green) and nuclear (DAPI, blue) staining of Raw264.7 cells. EVs and CeO_2_-EV were co-incubated with Raw264.7 cells for 12 h. Bar: 20 μm. (B) Fluorescence of CeO_2_ (FITC, green) and F-actin cytoskeleton (Phalloidin, red) and nuclear (DAPI, blue) staining of Raw264.7 cells. EVs and CeO_2_-EV were co-incubated with Raw264.7 cells for 12 h. Bar: 20 μm. Raw264.7 cells viability of EVs and CeO_2_ at varying concentrations using (C, D) CCK-8 assay and (E, F) Live/Dead staining. Bar: 50 μm. n = 3 per group. (G) Raw264.7 cells viability of CeO_2_-EV at varying concentrations using Live/Dead staining. Bar: 50 μm. Data are presented as Mean ± SD. **P* < 0.05, ***P* < 0.01; ns, no significance.


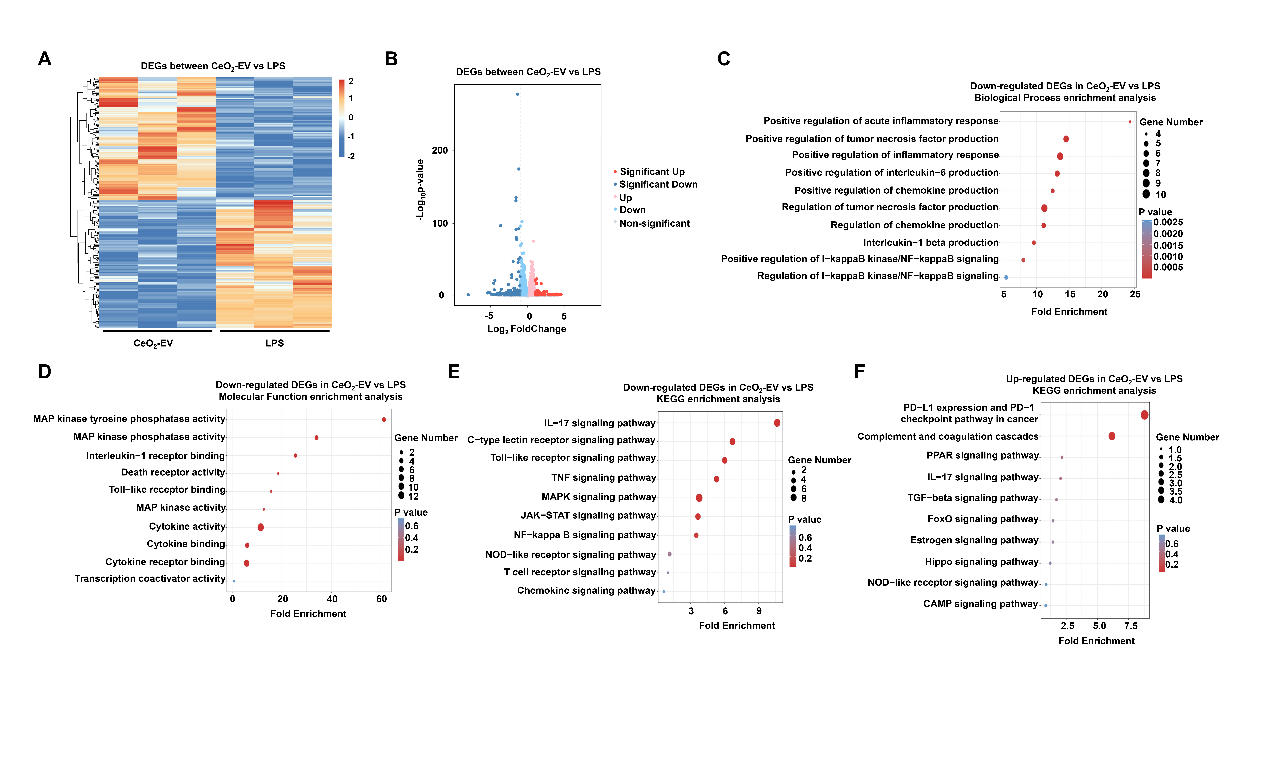


**Fig.S7.** RNA sequencing (RNA-seq) on LPS-pretreated RAW264.7 cells following CeO₂-EV treatment. (A) Heatmap showed the up-regulated and down-regulated differentially expressed genes (DEGs) (n = 3). (B) Volcano plots exhibited the DEGs in the CeO₂-EV compared to the LPS, including up-regulated DEGs (red) and down-regulated DEGs (blue). (C, D) Gene Ontology (GO) analysis of Biological Processes (BP) and Molecular Function (MF) enrichment analysis related to the down-regulated DEGs. (E, F) KEGG pathways enriched terms of down-regulated and up-regulated DEGs.


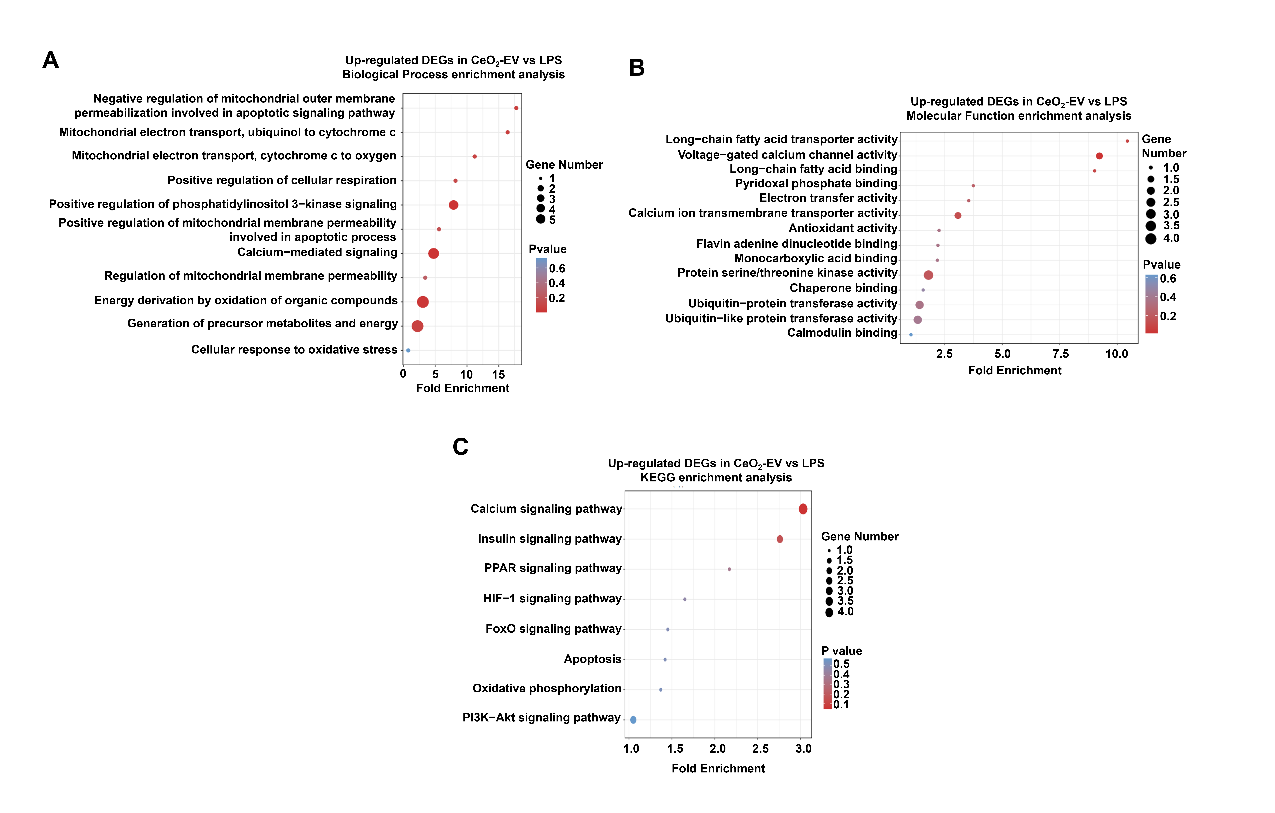


**Fig.S8.** The restoration of mitochondrial function on RNA-seq. (A, B) BP and MF enrichment analysis related to the up-regulated DEGs of mitochondrial related function. (C) KEGG pathways enriched terms of up-regulated DEGs in mitochondrial related function.


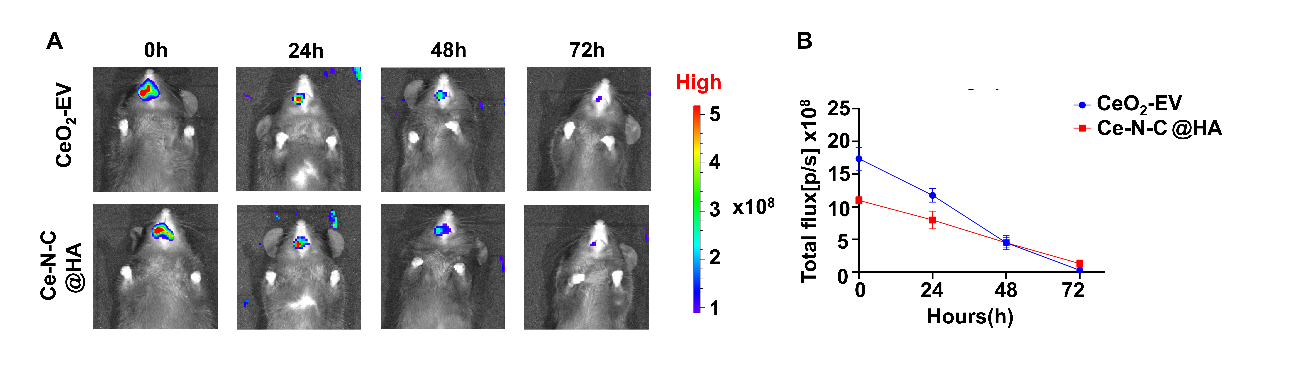


**Fig.S9.** In vivo distribution of Ce-N-C@HA and CeO₂-EV following local injection. (A, B) The distribution and fluorescence quantification of Ce-N-C@HA and CeO_2_-EV at 0 h, 24 h, 48 h and 72 h in the periodontal tissues. Data are presented as Mean ± SD.


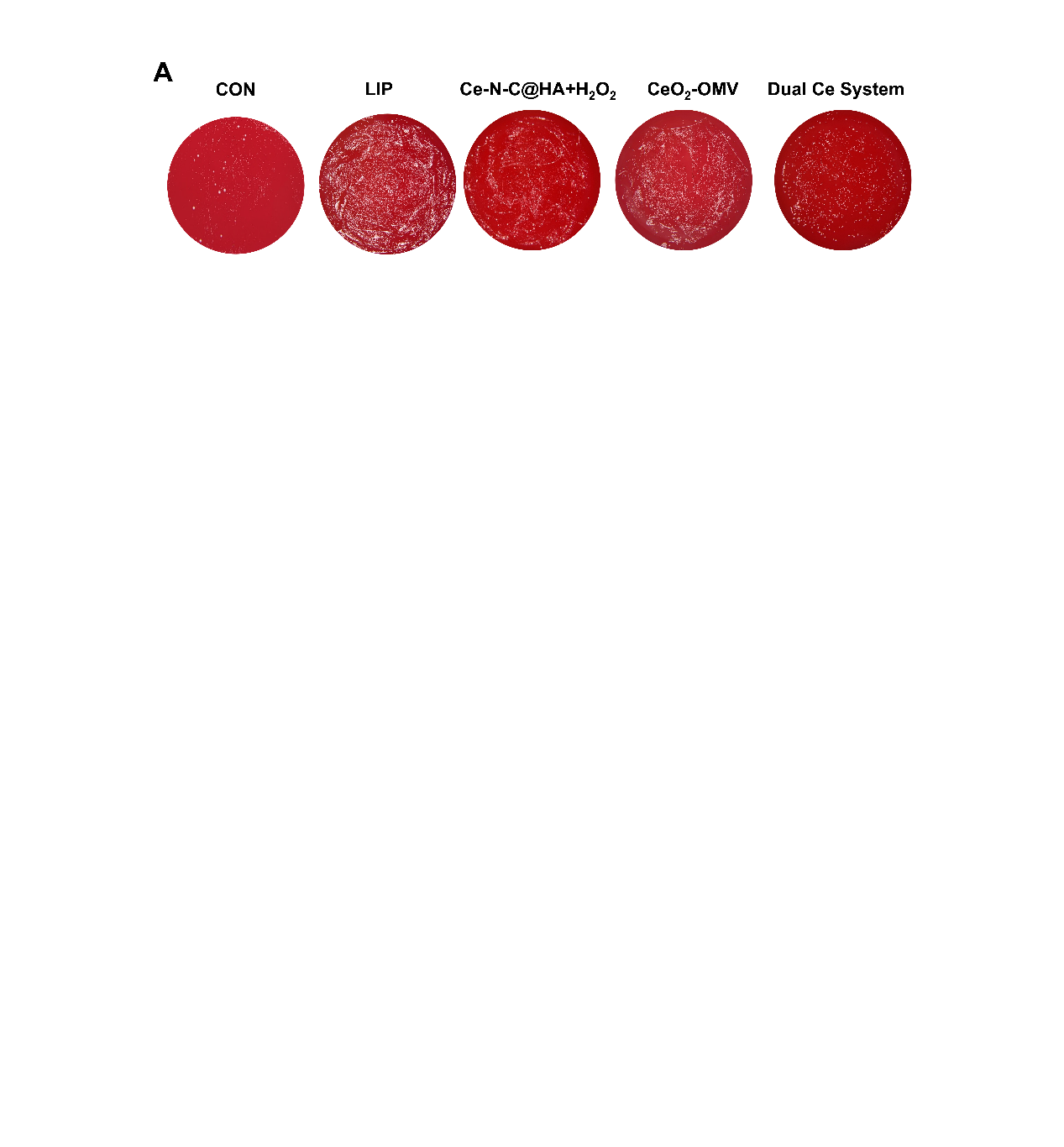


**Fig. S10.** Antibacterial test of Dual Ce System *in vitro*. (A) Culture of *P.gingivalis* from mice gingival crevicular fluid on TSA plates.


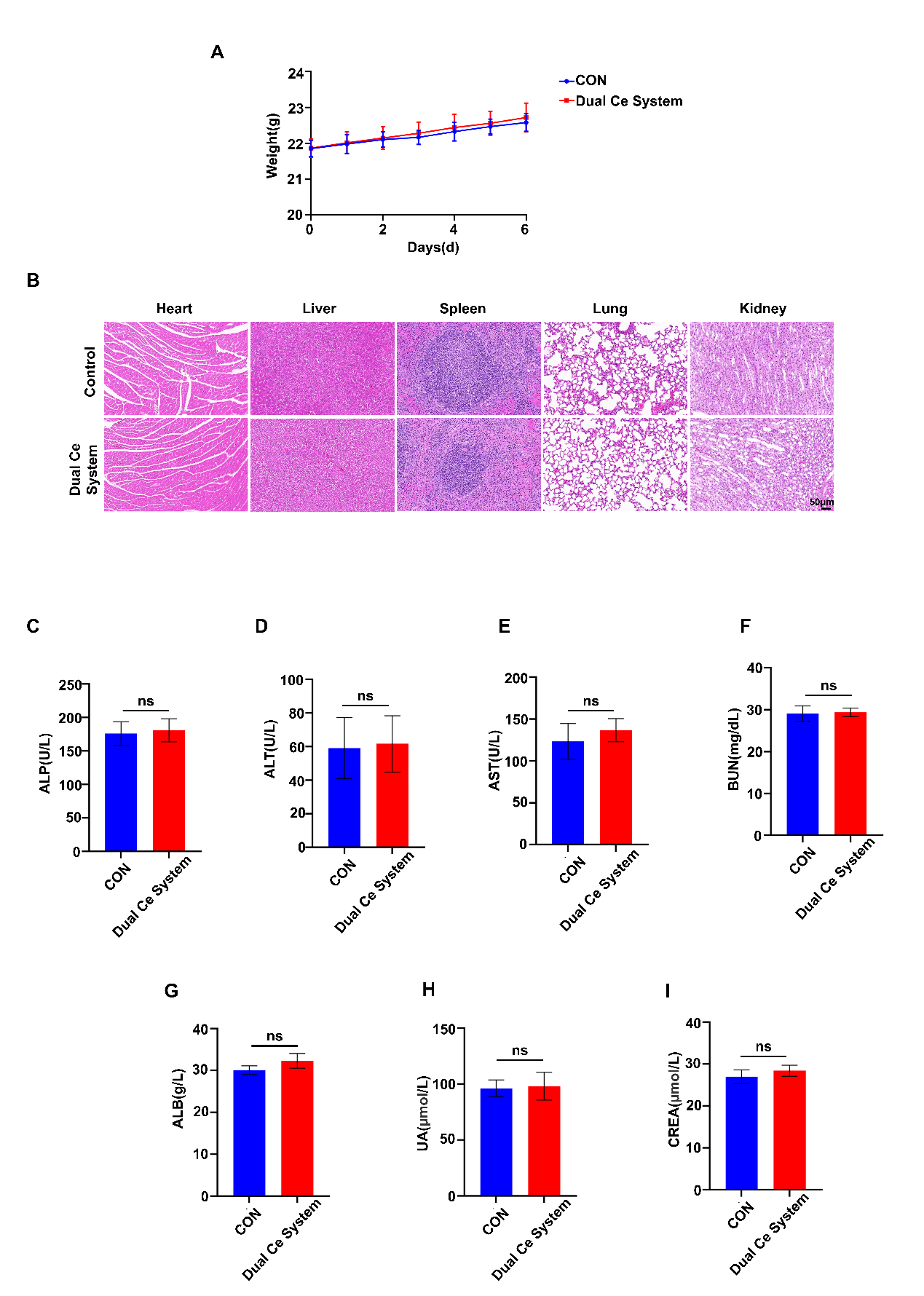


**Fig. S11.** Biosafety of the Dual Ce System. (A) Body Weight changes of mice within 0-6 d. (B) H&E staining of mice major organs. (C-I) Quantification of the mice liver biochemical parameters. Each mice received intravenous tail vein administration on Day 0. n = 3 per group. Data are presented as Mean ± SD. ns, no significance.
